# SIZ1‐Mediated SUMOylation of LBD29 Recruits ARF7 to Fine‐Tune Auxin Signaling in Lateral Root Development

**DOI:** 10.1002/advs.76633

**Published:** 2026-07-17

**Authors:** Jiaxuan Sui, Qianlan Yin, Yiying Chen, Liping Zhang, Xiuyuan Yu, Qianqian Yu, Zhaojun Ding, Xiangpei Kong

**Affiliations:** ^1^ The Key Laboratory of Plant Development and Environmental Adaptation Biology Ministry of Education Shandong Key Laboratory of Precision Molecular Crop Design and Breeding School of Life Science Shandong University Qingdao Shandong China; ^2^ State Key Laboratory of Microbial Technology Shandong University Qingdao Shandong China; ^3^ School of Life Sciences Liaocheng University Liaocheng Shandong China

**Keywords:** auxin signaling, lateral root, LBD29, SIZ1, SUMOylation

## Abstract

Auxin signaling orchestrates plant development through TRANSPORT INHIBITOR RESPONSE 1 (TIR1)/AUXIN‐SIGNALING F‐BOXs (AFBs)‐dependent nuclear auxin signaling pathway and emerging posttranslational modification (PTM) mechanisms. Here, we identify Small Ubiquitin‐like Modifier (SUMO) E3 ligase SAP AND MIZ1 DOMAIN‐CONTAINING LIGASE 1 (SIZ1)‐mediated SUMOylation as a critical regulator of auxin signaling. Auxin strongly induces SIZ1‐mediated SUMOylation, which reshapes the SIZ1 interactome and modulates large‐scale protein interactions. Transcriptome analysis shows that nearly 40% of Col‐0‐specific auxin‐responsive genes depend on *SIZ1*, and integrated RNA sequencing and immunoprecipitation–mass spectrometry reveal 103 genes that are co‐regulated at the transcriptional and protein levels. Among SUMOylation targets, the transcription factor LATERAL ORGAN BOUNDARIES DOMAIN 29 (LBD29) plays a key role in auxin‐mediated lateral root formation. SUMOylation promotes LBD29 ‐ AUXIN RESPONSE FACTOR 7 (ARF7) interaction, stabilizing a transcriptional activation complex that enhances downstream gene expression. Consistently, both *siz1‐2* and *siz1‐3* mutants show reduced auxin responsiveness and impaired lateral root development. Furthermore, *SIZ1* transcription is induced by auxin in an ARF7‐dependent manner, establishing a feedback loop. These findings establish a mechanistic framework in which SIZ1‐mediated SUMOylation links LBD29 function with ARF7‐dependent transcription, integrating PTM regulation into the auxin signaling network during lateral root development. This study highlights how dynamic protein modifications fine‐tune auxin signaling to coordinate developmental plasticity.

## Graphical abstract

Our findings establish SUMOylation as a key post‐translational control layer in auxin signaling. We show that SIZ1‐mediated SUMOylation of LBD29 promotes its association with ARF7, stabilizing a transcriptional hub that governs lateral root development. This mechanism generates a positive feedback loop—driven by ARF7‐dependent *SIZ1* transcription—that amplifies auxin signaling to precisely control root system architecture.

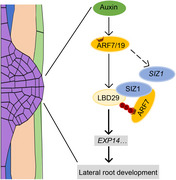

## 1 Introduction

Auxin is a pivotal regulator of nearly all aspects of plant growth and development [1, 2, 3]. Following the discovery of the TRANSPORT INHIBITOR RESPONSE 1 (TIR1) auxin receptor, understanding of auxin signal transduction has improved significantly [4, 5]. The nuclear‐localized signaling pathway is the most comprehensively studied framework and features three core components: the TIR1/AUXIN‐SIGNALING F‐BOX (AFB) receptor family, AUXIN OR INDOLE‐3‐ACETIC ACID (AUX/IAA) transcriptional repressors, and AUXIN RESPONSE FACTORs (ARFs) [3, 6]. Upon perception by TIR1/AFB receptors, auxins promote the ubiquitination‐dependent degradation of AUX/IAA repressors, thereby releasing ARFs from inhibition and enabling them to regulate the transcription of downstream target genes, ultimately mediating auxin responses. TIR1 also acts as an adenylate cyclase, which catalyzes the production of the second messenger cyclic AMP to mediate auxin signaling [7].

Beyond the TIR1/AFBs‐dependent auxin signaling pathway, recent studies have uncovered multiple alternative mechanisms of auxin perception and response [8, 9, 10]. Many of these pathways are regulated by posttranslational modifications (PTMs). PTMs, including protein phosphorylation, ubiquitination, and SUMOylation, regulate protein stability, enzymatic activity, subcellular localization, and interactions with other proteins or co‐factors [11]. In *Arabidopsis* roots, IAA treatment induces the rapid phosphorylation of approximately 1,000 proteins within just 2 min [12], a process dependent on AUXIN‐BINDING PROTEIN 1 (ABP1) and auxin‐activated transmembrane kinases (TMKs) [13, 14]. Under high‐auxin conditions, TMK interacts with ABP1/ABP1‐like (ABL) proteins to perceive auxin signaling and initiate downstream signaling through the activation of Rapidly Accelerated Fibrosarcoma (RAF)‐like protein kinases [8, 15, 16]. Subsequent proteolytic cleavage of TMK1 releases its C‐terminal kinase domain, which translocates into the nucleus and phosphorylates the non‐canonical Aux/IAA proteins, IAA32 and IAA34. This phosphorylation enhances the stability of Aux/IAAs and represses ARF‐mediated transcription [17]. Auxin‐activated TMK4 phosphorylates TRYPTOPHAN AMINOTRANSFERASE OF ARABIDOPSIS 1 (TAA1), inhibiting its auxin biosynthetic activity and establishing a negative feedback loop [18].

Lateral roots (LRs) are postembryonic organs that increase the surface area of the root system [19, 20]. Their primary functions involve soil anchorage and absorption of water and minerals, thereby providing fundamental support for plant growth and development [19, 21]. LR development can be divided into four key stages: founder cell specification [22], LR primordium (LRP) initiation [22, 23], primordium growth and morphogenesis [24], and LR emergence (LRE) and maturation [25].

During this complex developmental process, auxin plays a crucial regulatory role at all stages of LR formation. During LRP development, the ARF7 and ARF19 mediate auxin signaling through the transcriptional regulation of different downstream target genes [26, 27, 28]. Among these, the LATERAL ORGAN BOUNDARIES DOMAIN (LBD) family of proteins is a key downstream target that primarily promotes LR formation during the emergence stage by regulating *EXPANSIN* gene expression and cell wall remodeling. The mitogen‐activated protein kinase (MAPK) cascade‐mediated phosphorylation plays a crucial role in auxin signaling. The MKK4/5‐MPK3/6 cascade acts downstream of TMK1/4 and INFLORESCENCE DEFICIENT IN ABSCISSION (IDA)‐HAESA (HAE)/HAESA‐LIKE2 (HSL2) to regulate LR development by influencing cell division and cell wall remodeling, respectively [29, 30]. In addition, auxin‐activated MPK3/6 phosphorylates the cell cycle factor DPa, enhancing its stability and maintaining root stem cell identity [31]. MPK14 and VH1‐INTERACTING Kinase (VIK) are also involved in auxin‐induced LR development through phosphorylation of Ethylene Responsive Factor13 (ERF13) [32], which promotes its ubiquitination and degradation via MOS4‐ASSOCIATED COMPLEX 3A (MAC3A)/3B [33, 34]. Collectively, these findings highlight the central role of phosphorylation and other PTMs in fine‐tuning auxin signaling pathways during LR development.

Small Ubiquitin‐like Modifier (SUMO) proteins are ubiquitin‐like polypeptides (∼11 kDa) whose covalent attachment to substrates parallels ubiquitination. SUMOylation is mediated by an ATP‐dependent enzymatic cascade comprising heterodimeric E1 activating enzyme (SAE1/SAE2), E2 conjugating enzyme (SCE1), and E3 ligases [35, 36]. SUMO E3 ligases catalyze substrate‐specific SUMO conjugation by enhancing E2‐substrate interactions. In *Arabidopsis*, only two SUMO E3 ligases have been identified: SAP AND MIZ1 DOMAIN‐CONTAINING LIGASE 1 (SIZ1) and METHYL METHANE SULFONATE SENSITIVITY 21/HIGH PLOIDY 2 (MMS21/HPY2) [37]. SUMOylation is a key regulatory mechanism involved in plant abiotic stress adaptation. For example, SUMOylation of ARF7 suppresses its transcriptional activity during drought stress, thereby restricting LR formation as a part of drought‐induced root plasticity [38]. The *mms21* mutant exhibited impaired primary root development and altered expression of downstream auxin‐responsive genes [39, 40]. Another study revealed that MMS21 regulates root system architecture through the SUMOylation of IAA17, which stabilizes IAA17 by interfering with its TIR1‐mediated degradation [41]. Taken together, these studies highlight SUMOylation as a potentially critical regulator of auxin signal transduction. However, the precise molecular roles and mechanisms of auxin signaling remain unclear.

To further elucidate the role of SUMOylation in auxin signaling and uncover the downstream regulatory factors of SIZ1 involved in auxin‐induced LR formation, we performed immunoprecipitation–mass spectrometry (IP–MS) assays using *SIZ1*‐overexpressing seedlings. This analysis revealed numerous SIZ1‐interacting proteins upon auxin treatment, including well‐established components of the auxin signaling pathways. Notably, auxin was found to influence the substrate preference for SIZ1‐mediated SUMOylation. Complementary transcriptome profiling demonstrated that nearly 40% of auxin‐responsive genes required SIZ1 for proper expression. Using LBD29 as a representative case, we further illustrated how SUMOylation fine‐tuned auxin responses during LR development, underscoring the pivotal role of SUMOylation in auxin signaling and plant developmental regulation.

## 2 Results

### 2.1 SIZ1‐Mediated SUMOylation as a Key Regulator of Auxin Signaling

**FIGURE 1:**
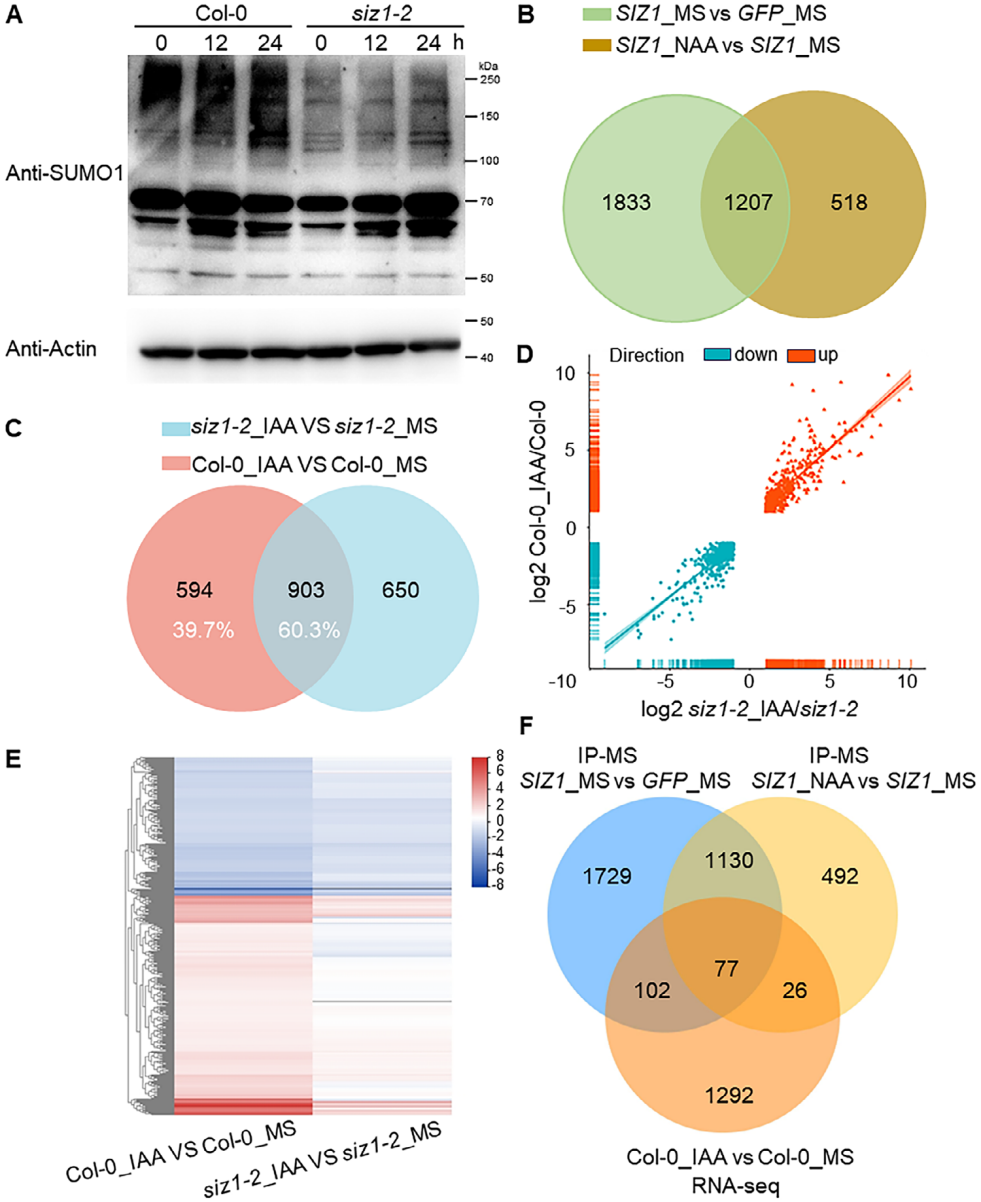
SIZ1 is involved in the auxin signal transduction pathway. (A) NAA induces SUMO1/2 conjugate accumulation that is facilitated by SIZ1. After treatment with 10 µM NAA for 0, 12, and 24 h, total proteins were extracted from 10‐d‐old Col‐0 and *siz1‐2* seedlings' roots and subjected to SDS‐PAGE. Then, the immunoblot analysis of anti‐SUMO1 antibody was performed. Actin was used as the loading control. The experiments were repeated independently three times with similar results. (B) Venn diagram shows the number of differential proteins between those immunoprecipitated from *Pro35S:SIZ1‐GFP* before and after 10 µM NAA treatment. |log^2^|≥ 1. (C) Venn diagram to show the number of differentially regulated genes in Col‐0 and *siz1‐2* before and after 0.5 µM IAA treatment. (D) The dot plot shows genes that are partially dependent on *SIZ1* among the 903 genes. The y‐axis represents the expression levels of genes induced by IAA in Col‐0, and the x‐axis represents the expression levels of these genes induced by IAA in *siz1‐2*. (E) The cluster heatmap shows a cluster analysis of 594 genes dependent on *SIZ1* after auxin treatment. (F) The Venn diagram shows the quantitative relationship between differentially expressed genes at the transcriptional level in Col‐0 and differentially expressed genes at the protein level in *Pro35S:SIZ1‐GFP* before and after auxin treatment.

To explore the role of SUMOylation in auxin signaling, we first examined the SUMOylated protein levels after NAA treatment. As shown in Figure 1A, the SUMO‐protein conjugate level accumulated significantly after 10 µM NAA treatment in Col‐0 seedling roots, whereas this response was markedly reduced in the *siz1‐2* mutant. Because SIZ1 functions as an E3 SUMO ligase at PTMs, we performed IP–MS analysis using the roots of *Pro35S:SIZ1‐GFP* and *Pro35S:GFP* seedlings with or without NAA addition. This analysis identified 3,040 proteins interacting with SIZ1 (Figure 1B), including known SIZ1‐binding partners such as SAE1A/B [42] and SCE1 [42], as well as the reported interactors TOPLESS‐RELATED 1 [43] and SENSITIVE TO PROTON RHIZOTOXICITY 1 (STOP1) [44], which served as critical positive controls under our experimental conditions (Table S1). Comparative analysis of SIZ1‐interacting proteins following NAA treatment revealed that the interaction between SIZ1 and 1,725 proteins was modulated by auxin (Figure 1B). In conclusion, these results indicated that SIZ1 plays a key role in regulating auxin signaling through protein SUMOylation.

To further investigate the role of SIZ1 in the auxin signal transduction pathway, we performed RNA sequencing (RNA‐seq) analysis of Col‐0 and *siz1‐2* mutant seedling roots before and after auxin treatment. Transcriptomic profiling identified 1497 differentially expressed genes (DEGs) in Col‐0 plants under IAA treatment, with 903 DEGs (60.3%) shared between Col‐0 and *siz1‐2* (Figure 1C). Dot map analysis showed that the 903 overlapping DEGs were partially dependent on *SIZ1* expression (Figure 1C,D). Among Col‐0‐specific DEGs, 594 (39.7%) were completely dependent on *SIZ1* (Figure 1C,E). Associative analysis of the RNA‐seq and IP–MS datasets revealed 103 genes that were co‐regulated by SIZ1 at both the transcriptional and protein levels in response to auxin treatment (Figure 1F).

Together, auxin‐induced SUMOylation dynamics combined with interactome and transcriptome analyses demonstrated that SIZ1‐mediated SUMOylation plays an important role in auxin signaling responses in root systems.

### 2.2 SIZ1‐Mediated Auxin Signaling Positively Regulates LR Development

**FIGURE 2:**
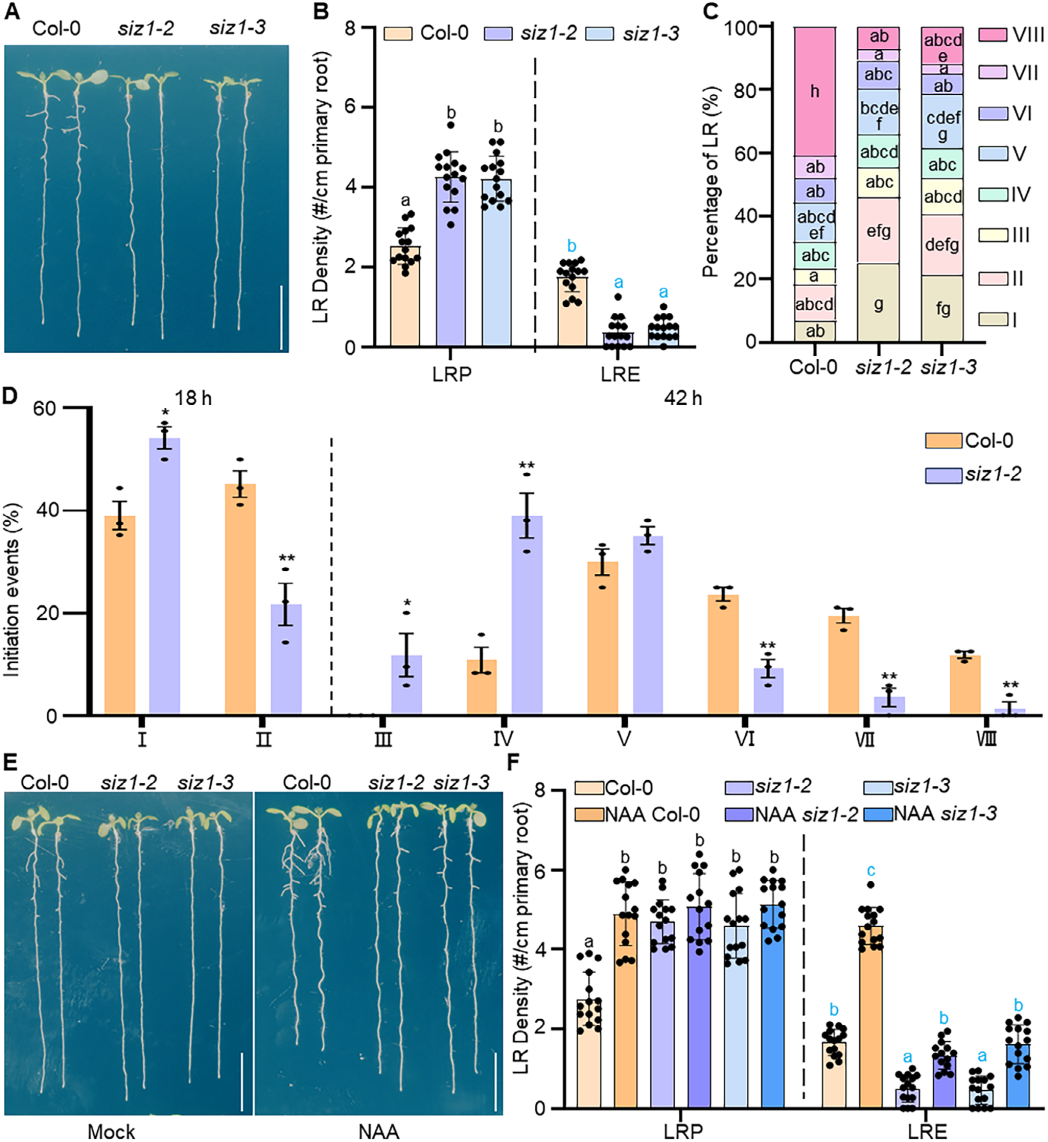
SIZ1 positively regulates auxin‐mediated LR development. (A) LR phenotypes were assessed in 8‐d‐old seedlings of Col‐0, *siz1‐2*, and *siz1‐3*. Scale bar, 1 cm. (B) LR density of Col‐0, *siz1‐2* and *siz1‐3*. LR density refers to the ratio of the number of LRs to primary root length. LRP and LRE represent LR primordia and emerged LR, respectively. Three independent biological replicates produced similar results, and at least 15 seedlings per line were used for statistical analysis. Different letters indicate significant differences in the same developmental stage LR proportion among different plants used one‐way ANOVA (*p* < 0.05, *n* ≥ 15). (C) The proportion of LR at different stages was analyzed in 8‐days‐old seedlings of Col‐0, *siz1‐2* and *siz1‐3*. Roman numerals I‐VIII represent LR developmental stages. Different letters indicate significant differences in the same developmental stage LR proportion among different plants using one‐way ANOVA (*p* < 0.05, *n* ≥ 15). (D) The synchronized initiation of LRP was induced using gravitropic stimulation at the site of root bending in the 3‐day‐old seedlings of Col‐0 and *siz1‐2* (∼30 seedlings were measured in each material). Phenotypic analysis of LRP stage I and stage II, stage III to E was achieved after 18 and 42 h of gravistimulus compared with Col‐0, respectively. As determined by a Student's *t* test. ^*^ *p* < 0.05, ^**^ *p* < 0.01. Data are indicated as means ± SE (*n* = 3). (E) The LR phenotypes were assessed in 8‐d‐old seedlings of both Col‐0, *siz1‐2* and *siz1‐3*, grown on ^1/2^MS medium or medium supplemented with 50 nM NAA. Scale bar, 1 cm. (F) LR density of the seedlings shown in (D). Three independent biological replicates produced similar results, and at least 15 seedlings per line were used for statistical analysis. Different letters indicate significant differences in the same developmental stage LR proportion among different plants used one‐way ANOVA (*p* < 0.05, *n* ≥ 15).

To investigate the biological significance of SIZ1 in auxin signaling, we performed a phenotypic analysis and found that both *siz1‐2* and *siz1‐3* exhibited a significant increase in LRP (stages I‐VII) but a notable decrease in LRE (stage VIII) compared to Col‐0 (Figure 2A,B), whereas the total number of LR showed no difference from that of Col‐0. We also analyzed the percentage of LRs at stages I‐VIII in *siz1* mutants and found that, compared to Col‐0, the *siz1* mutants exhibited delayed development at various stages of LR development (Figure 2C). We further conducted LR developmental profiling of *siz1‐2* mutants using gravistimulation assays. Comparative stage analysis revealed developmental arrest in *siz1‐2*, characterized by prolonged retention at stage I (elevated ratio) and attenuated progression to stage II (diminished ratio) 18 h post‐stimulation (Figure 2D). Extended temporal profiling (42 h) further demonstrated that the LR in *siz1‐2* accumulated at the early initiation phases (stages III‐IV), whereas Col‐0 LRs predominantly reached maturation stages (VI, VII, and VIII) (Figure 2D).

To confirm that the reduced LR phenotype in *siz1‐2* was caused by the loss of *SIZ1* function, we introduced *ProSIZ1:SIZ1‐GFP* into *siz1‐2* mutants and analyzed the LR phenotypes. The results showed that *siz1‐2/ProSIZ1:SIZ1‐GFP‐9* and *siz1‐2/ProSIZ1:SIZ1‐GFP‐10* completely restored the reduced LRE phenotype of *siz1‐2* (Figure S1), confirming that these phenotypes were caused by the *SIZ1* mutation. These results suggested that SIZ1 positively regulates LR development.

Next, we investigated the SIZ1 protein distribution during LR development in *siz1‐2/ProSIZ1:SIZ1‐GFP‐9* transgenic plants. Confocal microscopy revealed SIZ1 protein accumulation across all developmental stages of LRs (Figure S2). To elucidate whether SIZ1 is involved in auxin‐regulated LR development, we examined the LR phenotypes of Col‐0, *siz1‐2*, and *siz1‐3* mutant seedlings after NAA treatment. Both genotypes exhibited increased LR formation after treatment with 50 nM NAA. However, *siz1* mutants exhibited a hyposensitive response compared to Col‐0 plants (Figure 2E,F).

We also examined the primary root growth in the *siz1‐2* mutant, *SIZ1*‐overexpressing plants, and Col‐0 following NAA treatment. The results showed that *siz1‐2* displayed weaker NAA‐induced root growth inhibition than Col‐0. In contrast, *SIZ1*‐overexpressing plants did not exhibit enhanced auxin sensitivity (Figure S3).

In summary, these findings demonstrated that, in addition to regulating LR development, SIZ1‐mediated SUMOylation is also involved in auxin‐mediated primary root growth.

### 2.3 SIZ1 Interacts With LBD29 In Vitro and In Vivo

**FIGURE 3:**
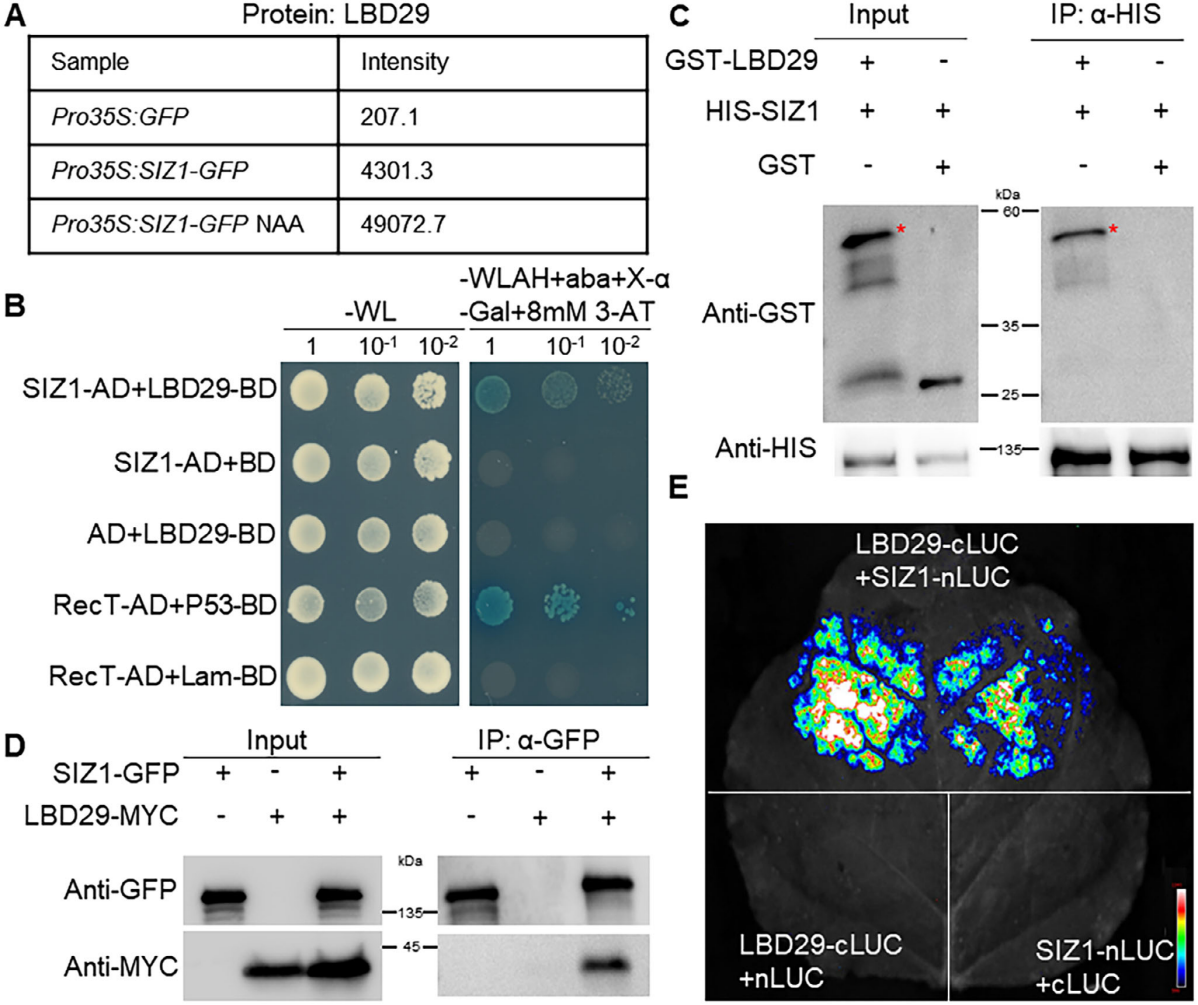
SIZ1 interacts with LBD29. (A) The sequence intensity of LBD29 identified by IP‐MS using 10‐d‐old *Pro35S:SIZ1‐GFP* and *Pro35S:GFP* seedlings before and after NAA treatment. (B) Interaction of SIZ1 and LBD29 in a yeast two‐hybrid (Y2H) assay. Interaction is indicated by the ability of cells to grow on SD/‐Trp‐Leu (‐WL) medium and SD/‐Trp‐Leu‐Ade‐His (‐WLAH) + X‐α‐gal + 8 mM 3‐AT medium for 3 days after plating. The negative control and positive control were transformed with commercial plasmids. AD, activation domain; BD, DNA‐binding domain. (C) In vitro pull‐down assay to verify the interaction between SIZ1 and LBD29. Recombinant GST‐LBD29 and HIS‐SIZ1, as well as GST protein, were purified from *Escherichia coli*. Immunoblot assays using anti‐GST and anti‐HIS antibodies were performed. (D) Co‐immunoprecipitation (co‐IP) assay in *Arabidopsis* protoplasts validating the interaction between LBD29 and SIZ1. *SIZ1‐GFP*, *LBD29‐MYC*, and *SIZ1‐GFP LBD29‐MYC* plasmids were transferred into the protoplasts of the free leaves of Col‐0, respectively. Immunoprecipitated using an anti‐GFP antibody, and the co‐immunoprecipitated LBD29‐MYC protein was detected using an anti‐MYC antibody. (E) Luciferase complementation imaging (LCI) assay performed in *N. benthamiana* leaves monitored the SIZ1 interacts with LBD29. Three different combinations, including two negative controls, were separately injected into four different areas of the leaf. nLUC/cLUC, N‐terminal/C‐terminal luciferase.

To elucidate the molecular mechanisms by which SIZ1 positively regulates the auxin signaling pathway, we further analyzed 103 auxin‐responsive genes co‐regulated by SIZ1 at both the transcriptional and protein levels (Figure 1E; Table S2). This analysis identified LBD29 (Figure 3A) as a transcription factor that critically regulates LR development in response to auxin. The yeast two‐hybrid (Y2H) assay also showed that SIZ1 and LBD29 exhibited strong interactions (Figure 3B). The in vitro pull‐down assay showed that SIZ1 directly interacted with LBD29 (Figure 3C). We performed a coimmunoprecipitation (co‐IP) assay using *Arabidopsis* protoplasts co‐expressing *SIZ1‐GFP* and *LBD29‐MYC* constructs. The results demonstrated co‐immunoprecipitation of SIZ1‐GFP with LBD29‐MYC (Figure 3D). Additionally, a luciferase complementation (LCI) assay confirmed the interaction between SIZ1 and LBD29 (Figure 3E). These results demonstrated that SIZ1 interacts with LBD29. To investigate whether auxin enhances the interaction between SIZ1 and LBD29, we performed Y2H and co‐IP assays. The results showed that auxin slightly promoted the interaction between SIZ1 and LBD29 (Figure S4A,B). In addition, auxin treatment led to the accumulation of LBD29 (Figure S4C).

LBD29 belongs to the LBD gene family, which contains an LOB domain, a conserved sequence responsible for DNA binding and protein‐protein interactions [45]. To identify the interaction domain of LBD29, we divided the LBD29 protein into an N‐terminal region containing the LOB domain and a C‐terminal region. Y2H and LCI results demonstrated that SIZ1 interacts with the C‐terminal region of LBD29 (Figure S5).

### 2.4 SIZ1 SUMOylated LBD29 at Lysine 21 and Lysine 24

**FIGURE 4:**
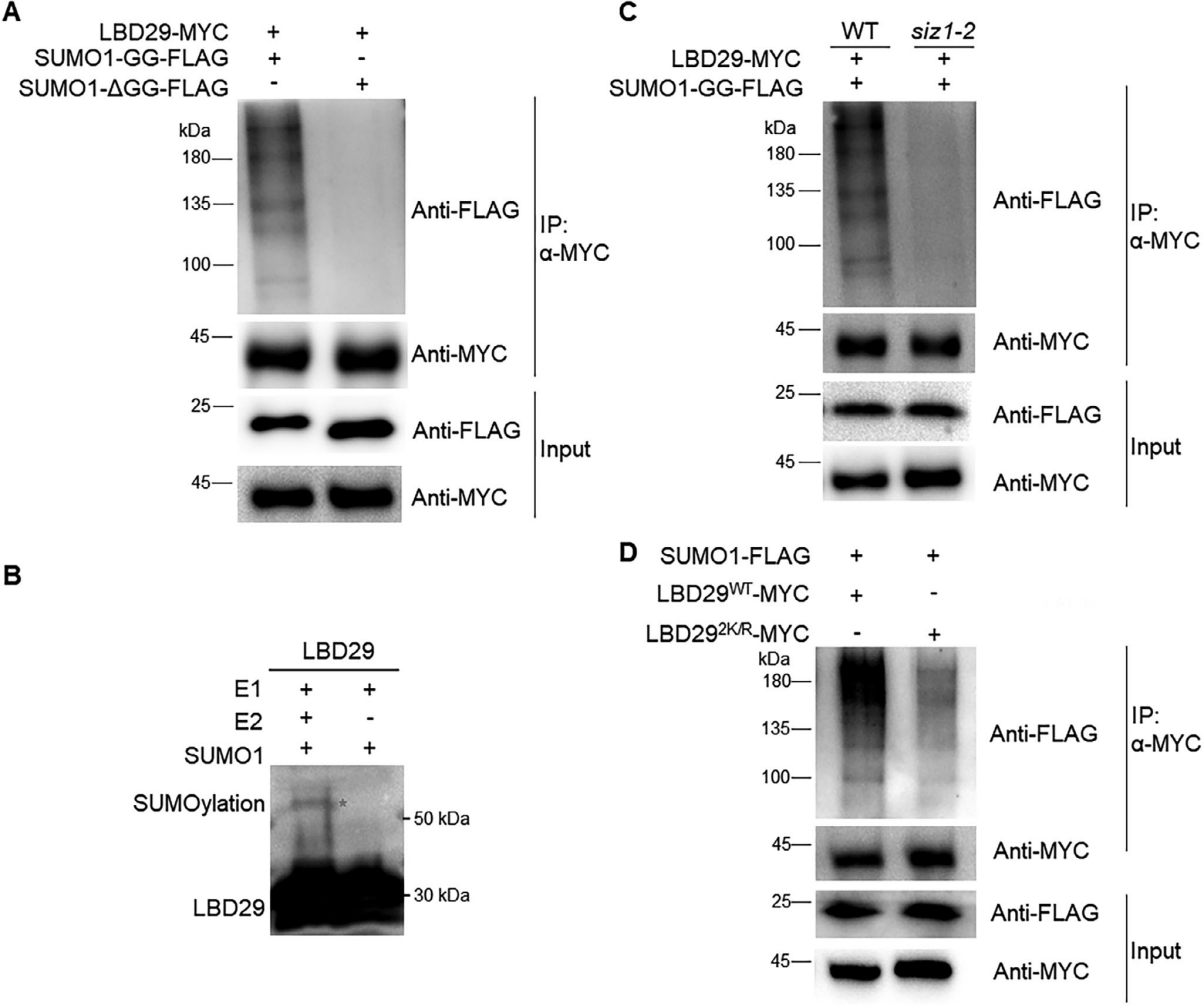
LBD29 is SUMOylated at Lys‐21 and Lys‐24 in vitro and in vivo. (A) LBD29 SUMOylation detected in the Col‐0 protoplasts. *LBD29‐MYC* was transiently co‐expressed with *FLAG‐SUMO1‐GG* or *FLAG‐SUMO1‐ΔGG* in Col‐0 protoplasts. Total protein extracts (input) were immunoprecipitated with anti‐MYC antibody. Anti‐MYC and anti‐FLAG antibodies were used for the immunoblotting analyses of the input proteins and the immunoprecipitated proteins. (B) In vitro SUMOylation detection experiment. The immunoblots of LBD29 that were modified by SUMO1. The LBD29 was fused with a Flag tag and co‐expressed with SUMO E1 and SUMO1 in the bacterial strain with or without SUMO E2 (SCE1) expression. An anti‐Flag antibody was used in the immunoblots to detect the LBD29 (30 kDa) and the SUMOylated forms (indicated by asterisks). (C) LBD29 SUMOylation detected in the Col‐0 and *siz1‐2* protoplasts. *LBD29‐MYC* was transiently co‐expressed with *FLAG‐SUMO1‐GG* in WT and *siz1‐2* protoplasts. Total protein extracts (input) were immunoprecipitated with anti‐MYC antibody. Anti‐MYC and anti‐FLAG antibodies were used for the immunoblotting analyses of the input proteins and the immunoprecipitated proteins. (D) The comparison of SUMOylation between LBD29^2K/R^ and wild‐type LBD29 in *Arabidopsis* protoplasts. The constructs *LBD29^2K/R^‐MYC*, *SUMO1‐FLAG*, and *LBD29‐MYC*, *SUMO1‐FLAG* were co‐transformed into *Arabidopsis* protoplasts, respectively. Proteins were immunoprecipitated using an anti‐MYC antibody, and the precipitated complexes were analyzed by immunoblotting with anti‐MYC and anti‐FLAG antibodies.

Since SIZ1 possesses SUMO E3 ligase activity, we performed an in vivo SUMOylation assay. As shown in Figure 4A, when *LBD29‐MYC* was co‐transformed with *SUMO1‐GG*, the SUMOylated bands of LBD29 were observed. However, no SUMOylated band of LBD29 was detected when co‐transformed with *SUMO1‐ΔGG*. This result demonstrates that LBD29 can be modified using SUMO1. To further validate SUMO conjugation to the LBD29, we performed a reconstituted SUMOylation assay in *Escherichia coli* expressing *Arabidopsis* SUMOylation machinery. These results indicate that LBD29 was SUMOylated in the presence of E2 (Figure 4B). To further investigate whether SUMOylation of LBD29 is dependent on SIZ1, we conducted an in vivo SUMOylation assay using Col‐0 and *siz1‐2*. As shown in Figure 4C, SUMOylation of LBD29 was clearly observed in Col‐0 cells, whereas SUMO1 conjugation on LBD29 was completely abolished in *siz1‐2*, confirming that SIZ1 is essential for LBD29 SUMOylation.

To identify the specific SUMOylation sites on LBD29, we performed bioinformatics predictions using GPS‐SUMO, which identified lysine residues K21 and K24 as potential sites. Next, we constructed an LBD29^2K/R^ (K21R/K24R) construct and performed SUMOylation assays in vivo. The results showed that the SUMOylation bands were significantly decreased in LBD29^2K/R^, suggesting that these two residues are key SUMOylation sites (Figure 4D).

### 2.5 SIZ1‐Mediated SUMOylation Promotes the Transcriptional Activity of LBD29

It is well known that SUMOylation affects multiple aspects of protein function, including protein stability, subcellular localization, and enzymatic activity. Therefore, we generated the wild‐type *ProLBD29:LBD29^WT^‐GFP/lbd29* and the mutated *ProLBD29:LBD29^2K/R^‐GFP/lbd29* transgenic lines and performed protein degradation assays. The results showed that SUMOylation did not affect the stability of the LBD29 protein (Figure S6A). We then examined the subcellular localization of Pro35S:LBD29‐GFP and Pro35S:LBD29^2K/R^‐GFP via transient expression assays in protoplasts and found that SUMOylation did not alter the subcellular localization of LBD29 (Figure S6B).

**FIGURE 5:**
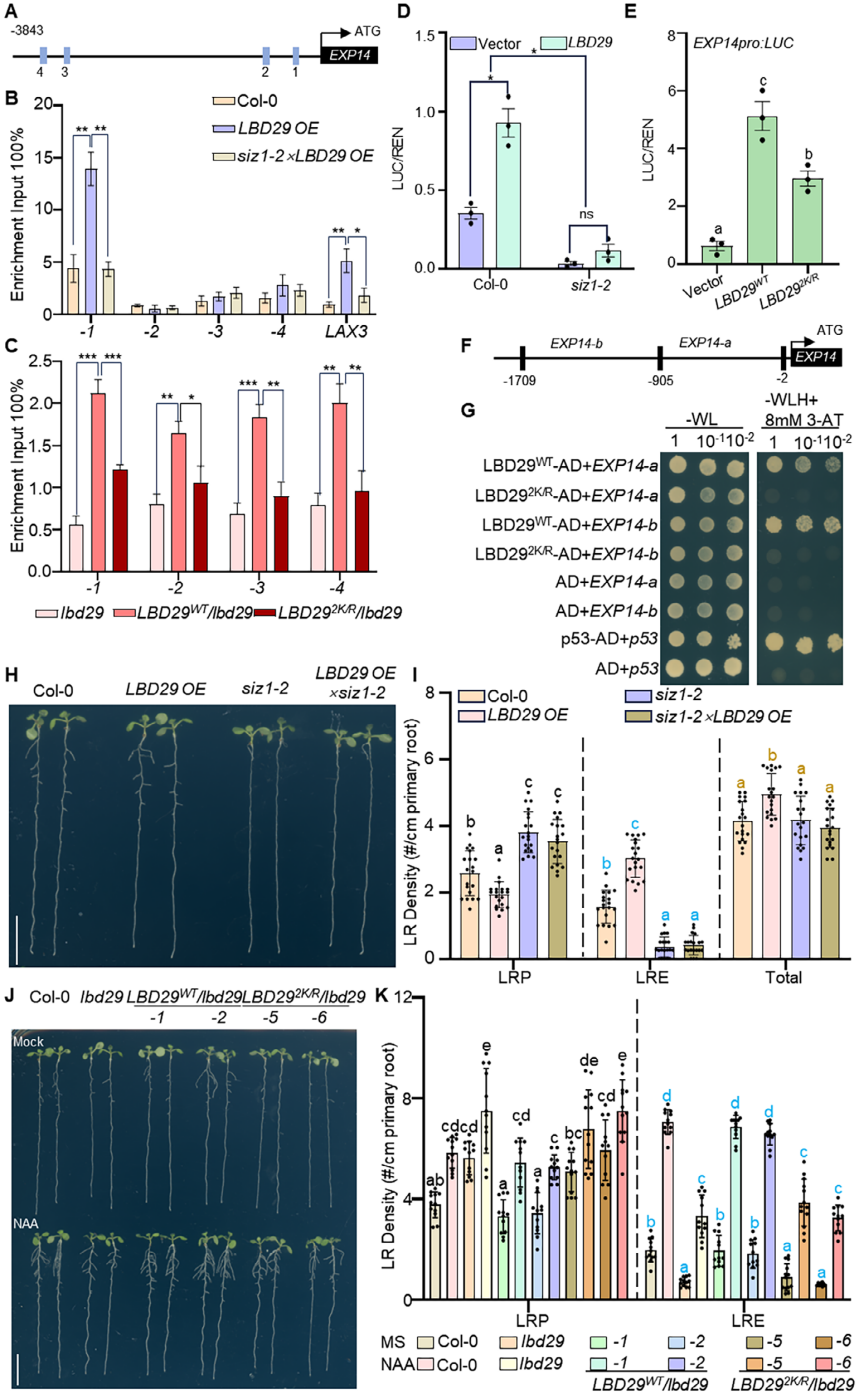
SIZ1‐mediated SUMOylation promotes the transcriptional activity of LBD29 in LR development. (A) Schematic of the *EXP14* promoter and the PCR amplicons (1‐4) used for chromatin immunoprecipitation (ChIP)‐qPCR. (B) ChIP‐qPCR results show the enrichment of LBD29 on the chromatin of *EXP14*. Sonicated chromatin from 10‐d‐old seedlings (Col‐0, *LBD29 OE* (*Pro35S:LBD29‐6Gly‐FLAG*), *siz1‐2×LBD29 OE*) was precipitated with anti‐FLAG antibodies. The precipitated DNA was used as a template for qPCR analysis, with primers targeting different regions of *EXP14*, as shown in (A). Three biological replicates with three technical replicates for each biological replicate were performed with similar results. ^**^ *p* < 0.01, ^*^ *p* < 0.05, as determined by a Student's *t* test. Data are represented as mean ± SE (*n* = 3). (C) ChIP‐qPCR results show the enrichment of LBD29 on the chromatin of *EXP14*. Sonicated chromatin from 10‐d‐old seedlings (*lbd19*, *LBD29^WT^/lbd29* (*ProLBD29:LBD29‐GFP^WT^/lbd29*), *LBD29^2K/R^/lbd29* (*ProLBD29:LBD29‐GFP^K21/24R^/lbd29*)) was precipitated with anti‐GFP antibodies. The precipitated DNA was used as a template for qPCR analysis, with primers targeting different regions of *EXP14*, as shown in (A). Three biological replicates with three technical replicates for each biological replicate were performed with similar results. ^***^ *p* < 0.001, ^**^ *p* < 0.01, ^*^ *p* < 0.05, as determined by a Student's *t* test. Data represent mean ± SE (*n* = 3). (D) The transcriptional activity of LBD29 on *ProEXP14:LUC* was detected in the Col‐0 or *siz1‐2* mutant protoplasts. The empty vector *pBI221* was used as a negative control. Three biological replicates with three technical replicates for each biological replicate were performed with similar results. *p < 0.05, as determined by a Student's *t* test. Data represent mean ± SE (*n* = 3). (E) The transcriptional activity of LBD29^WT^ and LBD29^2K/R^ on *ProEXP14:LUC* was detected in the Col‐0 protoplasts. The empty vector *pBI221* was used as a negative control. Three biological replicates with three technical replicates for each biological replicate were performed with similar results. Different lowercase letters indicate significant differences by one‐way ANOVA, followed by Tukey's multiple comparison test (*p* < 0.05). Data represent mean ± SE (*n* = 3). (F) Schematic diagram of the *EXP14* promoter used for yeast one‐hybrid (Y1H) assay. (G) Y1H assay testing binding of LBD29^WT^ and LBD29^2K/R^ to the *EXP14* promoter. The yeast transformants were dropped onto SD‐WL medium. The yeast transformants were dropped onto SD‐WLH + 8 mM 3‐AT medium. The negative control and positive control were transformed with commercial plasmids. (H) LR phenotypes were examined in 8‐d‐old seedlings, including Col‐0, *siz1‐2*, *LBD29 OE/*Col‐0, and *LBD29 OE/siz1‐2*. Scale bar, 1 cm. (I) LR density of Col‐0, *siz1‐2*, *LBD29 OE*, and *LBD29 OE/siz1‐2*. Three independent biological replicates produced similar results, and at least 18 seedlings per line were used for statistical analysis. Different lowercase letters indicate significant differences by one‐way ANOVA, followed by Tukey's multiple comparison test (*p* < 0.05). Data are indicated as means ± SD (*n* ≥ 18). (J) LR phenotypes were examined in 9‐d‐old seedlings of both Col‐0, *lbd29*, *LBD29^WT^/lbd29#1*, *LBD29^WT^/lbd29#2*, *LBD29^2K/R^/lbd29#5* and *LBD29^2K/R^/lbd29#6*, grown on ^1/2^MS medium or medium supplemented with 50 nM NAA. Scale bar, 1 cm. (K) LR density of the seedlings shown in (J). Three independent biological replicates produced similar results, and at least 12 seedlings per line were used for statistical analysis. Different lowercase letters indicate significant differences by one‐way ANOVA, followed by Tukey's multiple comparison test (*p* < 0.05). Data are indicated as means ± SD (*n* ≥ 12).

To explore whether SIZ1‐mediated SUMOylation of LBD29 affects its activity, we generated *Pro35S:LBD29‐FLAG*/*siz1‐2* transgenic plants by genetic crossing. During LR development, LBD proteins directly regulate the auxin influx carrier*s LIKE AUX1 3* (*LAX3*) and *EXPANSIN14* (*EXP14*) to promote LRE [46]. A chromatin immunoprecipitation‐qPCR (ChIP‐qPCR) assay using seedlings showed that the enrichment of *EXP14* and *LAX3* promoter regions in the *Pro35S:LBD29‐FLAG* line was markedly reduced in the *Pro35S:LBD29‐FLAG*/*siz1‐2* line (Figure 5A,B). We then examined the transcriptional activity of LBD29 on the expression of the *EXP14* promoter using transient expression assays in Col‐0 and *siz1‐2* leaf protoplasts and found that LBD29‐mediated induction of *EXP14* expression was completely abolished in *siz1‐2* mutants, as evidenced by decreased ProEXP14:LUC activity (Figure 5D).

To explore the impact of the K21 and K24 SUMOylation sites on the transcriptional activity of LBD29, we performed transient expression assays in *Arabidopsis* protoplasts. The results showed that compared to the wild‐type LBD29^WT^, the mutated LBD29^2K/R^ exhibited severely compromised transactivation potential toward *EXP14* (Figure 5E). Based on the predicted potential LBD29‐binding sites [47, 48], we divided the promoter of *EXP14* into two fragments (*EXP14‐a* and *EXP14‐b*). Yeast one‐hybrid (Y1H) analysis further confirmed that LBD29^2K/R^ lost its ability to bind DNA to the *EXP14* promoter (Figure 5F,G). ChIP‐qPCR revealed a markedly reduced enrichment of the *EXP14* promoter in *ProLBD29:LBD29^2K/R^‐GFP/lbd29* plants (Figure 5C). Additionally, Y2H assays revealed that, compared to *LBD29^WT^‐BD*, the *LBD29^2K/R^‐BD* construct completely lost its transcriptional autoactivation capacity (Figure S7). Collectively, these results demonstrated that SUMOylation is an essential PTM that enables LBD29 to target chromatin and execute its transcriptional functions.

### 2.6 SIZ1‐Mediated SUMOylation is Required for LBD29‐Induced LR Formation

LBD29 plays a crucial role in LR formation and is essential for maintaining the division capacity of pericycle cells [49]. Similar to the *siz1‐2* mutant, the *lbd29* mutant exhibited a significant increase in LRP and a decrease in LRE, whereas the total number of LR was comparable to that of Col‐0 (Figure S8A,B). We also analyzed the percentage of LRs at stages I ‐VIII in the *lbd29* mutant and found that it exhibited delayed LR development similar to *siz1* mutants (Figure S8C). In addition, gravistimulation assays revealed that all stages of LR formation in *lbd29* were delayed in *lbd29* relative to those in WT (Figure S8D). To investigate the genetic relationship between *SIZ1* and *LBD29*, we generated *siz1‐2/lbd29* double mutants. Phenotypic analysis revealed similar LRE defects in the single and double mutants without additive effects, suggesting *SIZ1* and *LBD29* function in the same pathway to promote LR formation (Figure S8E,F).

Since SIZ1‐mediated SUMOylation is important for LBD29 activity, we investigated its role in LR development. In the WT background, *Pro35S:LBD29‐FLAG* exhibited a significantly higher LRE density than Col‐0; however, the *Pro35S:LBD29‐FLAG*/*siz1‐2* lines showed a significantly reduced LRE density, similar to *the siz1‐2* mutant (Figure 5H,I). These results clearly indicated that SIZ1‐mediated SUMOylation was required for LBD29‐induced LR formation.

To further examine whether the SUMOylation at K21 and K24 of LBD29 regulates LR formation, we examined the LR phenotypes of *ProLBD29:LBD29^WT^‐GFP/lbd29* and *ProLBD29:LBD29^2K/R^‐GFP/lbd29*. The results showed that the WT version of *ProLBD29:LBD29‐GFP* (#1 and #2) fully rescued the LR developmental defects in the *lbd29* mutant (Figure 5J,K). However, mutant *ProLBD29:LBD29^2K/R^‐GFP* (#5 and #6) failed to rescue the *lbd29* mutant phenotype during LR development (Figure 5J,K). Importantly, after auxin treatment, *ProLBD29:LBD29‐GFP* lines (#1 and #2) exhibited a similar degree of LRE induction as Col‐0, whereas *ProLBD29:LBD29^2K/R^‐GFP* (#5 and #6) phenocopied the *lbd29* mutant in their auxin response (Figure 5J,K). These results indicated that SUMOylation at K21 and K24 is critical for LBD29 function in regulating LR development.

### 2.7 SUMOylation of LBD29 Enhances Its Interaction With ARF7

**FIGURE 6:**
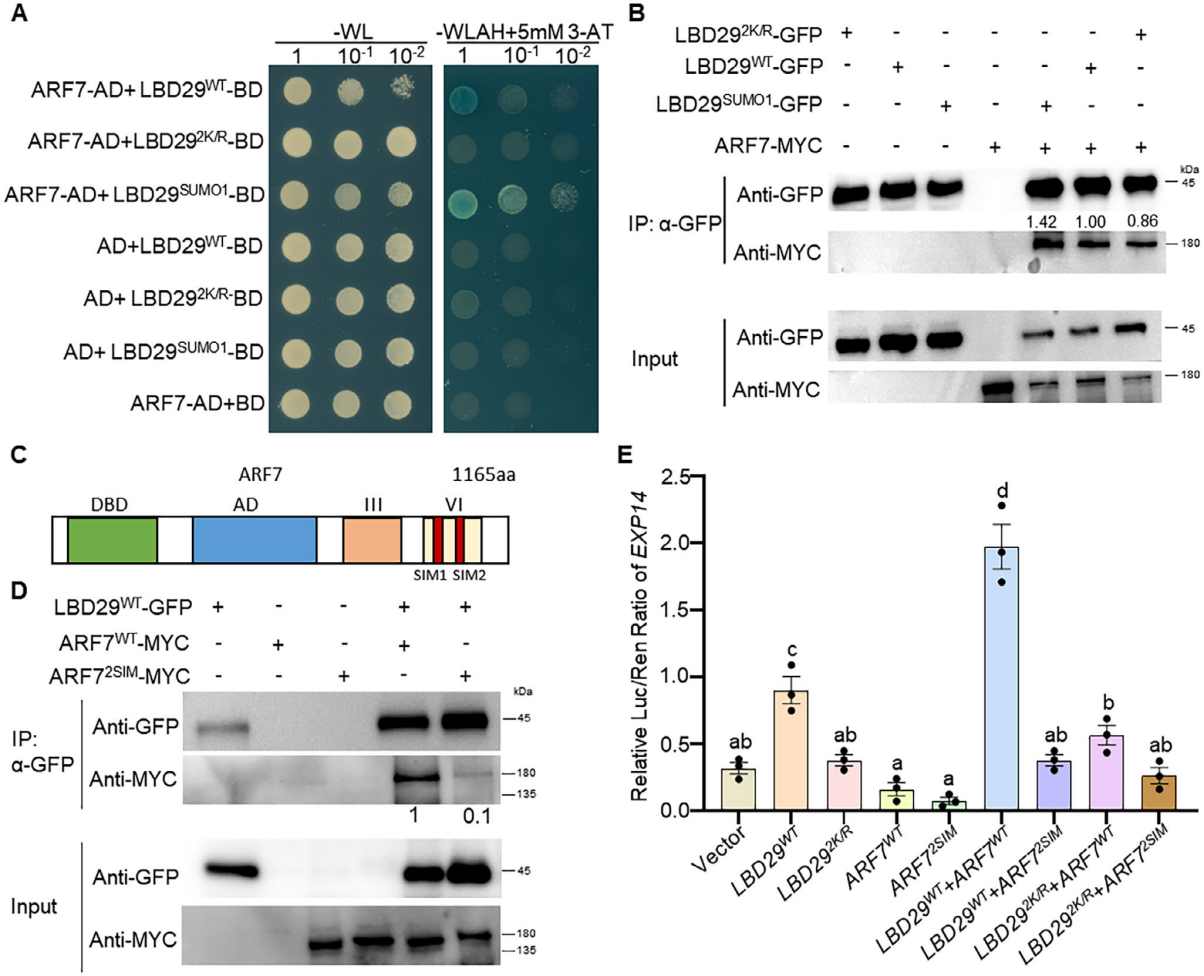
SUMOylation of LBD29 enhances its interaction with ARF7. (A) Interaction of LBD29^WT^, LBD29^2K/R^ or LBD29^SUMO1^ with ARF7 in a Y2H assay. Yeast cells were grown on SD‐WL and SD‐WLAH + 5 mM 3‐AT medium. Yeast cells co‐transformed with *pGBKT7‐LBD29^WT^*, *pGBKT7‐LBD29^2K/R^* or *pGBKT7‐LBD29^SUMO1^* derivatives (bait) and *pGADT7‐ARF7* were dropped onto SD‐WL and SD‐WLAH + 5 mM 3‐AT medium to assess interactions with ARF7. (B) Interaction of LBD29^WT^, LBD29^2K/R^, or LBD29^SUMO1^ with ARF7 in a co‐IP assay. Protein extracts (input) were immunoprecipitated with anti‐GFP antibody. Immunoblots were developed with anti‐GFP antibody to detect LBD29^WT^‐GFP, LBD29^2K/R^‐GFP, and LBD29^SUMO1^‐GFP and with anti‐MYC to detect ARF7‐MYC. (C) Diagrams of ARF7 showing putative SIMs. (D) Interaction of ARF7^WT^ or ARF7^2SIM^ with LBD29^WT^ in a co‐IP assay. Protein extracts (input) were immunoprecipitated with anti‐GFP antibody. Immunoblots were developed with anti‐GFP antibody to detect LBD29^WT^‐GFP and with anti‐MYC to detect ARF7^WT^‐MYC and ARF7^2SIM^‐MYC. The abundance of ARF7^WT^‐MYC relative to LBD29^WT^‐GFP was calibrated to 1.00. (E) Relative activity of the *ProEXP14:LUC* reporter in *Arabidopsis* protoplasts co‐transformed with the indicated effector constructs. Three biological replicates with three technical replicates for each biological replicate were performed with similar results. Different lowercase letters indicate significant differences by one‐way ANOVA, followed by Tukey's multiple comparison test (*p* < 0.05). Data represent mean ± SE (*n* = 3).

LBD18 enhances the transcriptional activity of ARF7 by competitively binding to the PB1 domain (Phox and Bem1 domains) of the repressor IAA14 [50]. Here, we also observed that LBD29^WT^ interacted with ARF7, and that this interaction was significantly strengthened by the SUMO‐mimic form of LBD29^SUMO1^, whereas the SUMOylation‐deficient mutant LBD29^2K/R^ exhibited a substantially weaker association with ARF7 (Figure 6A). Furthermore, a co‐IP assay confirmed that ARF7 exhibited a stronger interaction with LBD29^SUMO1^ but a weaker interaction with LBD29^2K/R^ (Figure 6B). These results suggest that SUMOylation of LBD29 enables the recruitment of ARF7. SUMO‐interacting motifs (SIMs) are short sequence elements that specifically recognize SUMO and mediate SUMO‐dependent protein‐protein interactions [51]. Sequence analysis of ARF7 revealed two putative SIMs located within domain IV (Figure 6C). To test their function, we generated the SIM1 (1097: ILLV→GGGG)/SIM2(1115: IKIL→GKGG) double mutation version of ARF7 (ARF7^2SIM^) and performed co‐IP assays. The results showed that ARF7^2SIM^ displayed a markedly reduced interaction with LBD29 (Figure 6D), supporting the idea that SUMOylation strengthens the LBD29–ARF7 association through SIM–SUMO recognition. Transient expression assays consistently showed that co‐expression of ARF7 significantly enhanced LBD29^WT^‐mediated activation of *EXP14* transcription, but this synergistic effect was abolished in the LBD29^2K/R^ mutant. Moreover, ARF7^2SIM^ completely abolished the transcriptional activation of LBD29 (Figure 6E). Taken together, these results demonstrate that ARF7 preferentially promotes the transcriptional activity of SUMOylated LBD29.

It has been previously shown that ARF7 interacts with MEDIATOR 25 (MED25) (a key Mediator subunit that bridges transcription factors and RNA polymerase II) [52], and Y2H assays showed no direct interaction between LBD29, LBD29^2K/R^ and MED25 (Figure S9). Therefore, SUMOylation of LBD29 may enhance transcriptional activity indirectly by strengthening its interaction with ARF7, which in turn recruits the Mediator complex via ARF7.

ARF7 can be SUMOylated under drought stress [38]. Notably, ARF7 was also identified among the proteins that co‐immunoprecipitated with SIZ1 (Figure S10A), and this interaction was further validated using a Y2H assay (Figure S10B). Next, we conducted an in vivo SUMOylation assay of ARF7. The results showed that ARF7 could not be SUMOylated under normal conditions, whereas the positive control, STOP1, showed obvious SUMOylation bands, which is consistent with previous studies [44] (Figure S10C). This suggests that SIZ1, LBD29, and ARF7 may function together as ternary complexes.

### 2.8 NAA Induces SIZ1 Transcription

Our findings demonstrated that SIZ1 positively regulates auxin signaling‐mediated LR formation. Therefore, we investigated whether auxins influence *SIZ1* expression. Quantitative Real‐time PCR (qRT‐PCR) analysis revealed that NAA treatment induces *SIZ1* transcription in a time‐dependent manner (Figure S11A). Consistent with the qRT‐PCR data, the NAA‐treated seedlings exhibited significantly stronger GFP fluorescence, particularly during the LRE stage (Figure S11B). In parallel, we examined the effect of auxin on the SIZ1 protein and found that it did not alter SIZ1 protein stability (Figure S12A,B).

The ARF7/19‐LBD16/18/29 signaling module is well known to play a crucial role in auxin‐mediated LR development [34]. To investigate whether NAA‐induced *SIZ1* transcription depends on this module, we examined *SIZ1* expression patterns in Col‐0, *arf7*, *arf7*/*arf19* double mutants, and *lbd16*/*18*/*29* triple mutants after NAA treatment. The results showed that, compared to Col‐0, auxin‐induced *SIZ1* expression was significantly attenuated in both *arf7* and *arf7/arf19* double mutants, but remained unaffected in *lbd16/18/29* triple mutants. These findings demonstrate that ARF7, but not downstream of LBD16/18/29, is required for auxin‐induced *SIZ1* transcription (Figure S11C). However, Y1H showed that ARF7 could not directly bind to the *SIZ1* promoter region, suggesting that ARF7 indirectly regulates *SIZ1* transcription (Figure S11D,E). In parallel, the SUMOylation assay of *N. benthamiana* leaves and *Arabidopsis* protoplasts demonstrated that NAA treatment enhanced the SUMOylation of LBD29, with SUMO conjugate accumulation increasing progressively over time (Figure S13A,B).

Since ARF7 is required for auxin‐induced *SIZ1* transcription, we investigated whether ARF7 affects the SUMOylation of LBD29. We examined the SUMOylation levels of LBD29 in Col‐0 and *arf7/arf19* double mutants. The results showed that under normal conditions, compared to Col‐0, the SUMOylation level of LBD29 did not change in *the arf7/arf19* double mutant. However, upon auxin treatment, SUMOylation of LBD29 was strongly induced in Col‐0, but this induction was significantly reduced in the *arf7/arf19* double mutant (Figure S13B). These results indicate that ARF7 is required for the auxin‐induced SUMOylation of LBD29.

## 3 Discussion

Auxin signaling has long been recognized as the central coordinator of plant development and has been traditionally studied through the nuclear pathway involving TIR1 receptors, ARFs, and Aux/IAAs. However, recent studies have reported multiple mechanisms. For example, TMKs and RAFs kinases modulate rapid auxin signaling [15, 16, 53], whereas FERONIA kinases predominantly regulate auxin‐induced root hair elongation [54]. VIK and MPK14 play crucial roles in auxin‐induced LR development [32, 34]. In addition, low water stress induced redox‐dependent IAA3 multimerization to regulate auxin‐controlled LR formation [55]. Collectively, these findings demonstrate that PTMs play an indispensable role in fine‐tuning auxin responses across various plant growth and developmental processes.

Among PTMs, SUMOylation has emerged as a particularly important regulatory mechanism in cellular signaling. SUMO proteins are a class of 11‐kDa ubiquitin‐like polypeptides that mediate reversible PTM through an enzymatic cascade involving E1 activation, E2 conjugation, and E3 ligation.

SIZ1, a key SUMO E3 ligase in *Arabidopsis*, regulates a wide range of developmental processes and stress responses, such as flowering time [56], defense responses [42, 43, 57, 58], and drought stress tolerance [59, 60]. Our study demonstrates that SIZ1 also regulates LR development, thereby revealing a new function of SIZ1 in root development. It was reported that *SIZ1* is expressed in multiple tissues, including LRP [59]. How a ubiquitously expressed enzyme specifically responds to auxin signals to control LR formation. We propose that, as a post‐translational modification, the functional specificity of SUMOylation does not entirely depend on the expression level of the enzyme but relies more on substrate availability, such as LBD29, which is dynamically regulated by auxin signaling. In addition, auxin signals can trigger transient interactions between SIZ1 and its targets, enabling signal‐dependent modification. Together, these mechanisms allow SIZ1 to precisely respond to auxin signals and regulate LR development.

Under normal growth conditions, SIZ1 preferentially modifies auxin signaling components such as LBD29, thereby promoting LR formation. However, under low phosphate (Pi) stress, the primary substrate of SIZ1 might shift from auxin‐related targets to PHOSPHATE STARVATION RESPONSE1 (PHR1), a master regulator of phosphate homeostasis [61]. By SUMOylating PHR1, SIZ1 suppresses LR branching under Pi deficiency. This context‐dependent substrate switching highlights the pleiotropic nature of SIZ1. It acts as a central regulatory hub that integrates two distinct signaling pathways: promoting root branching via auxin under normal conditions, while restraining root branching via PHR1 under Pi limitation. Thus, the same SUMO ligase shows opposite effects on LR development depending on environmental cues, reflecting a sophisticated regulatory mechanism that ensures optimal root architecture in response to nutrient availability.

**FIGURE 7:**
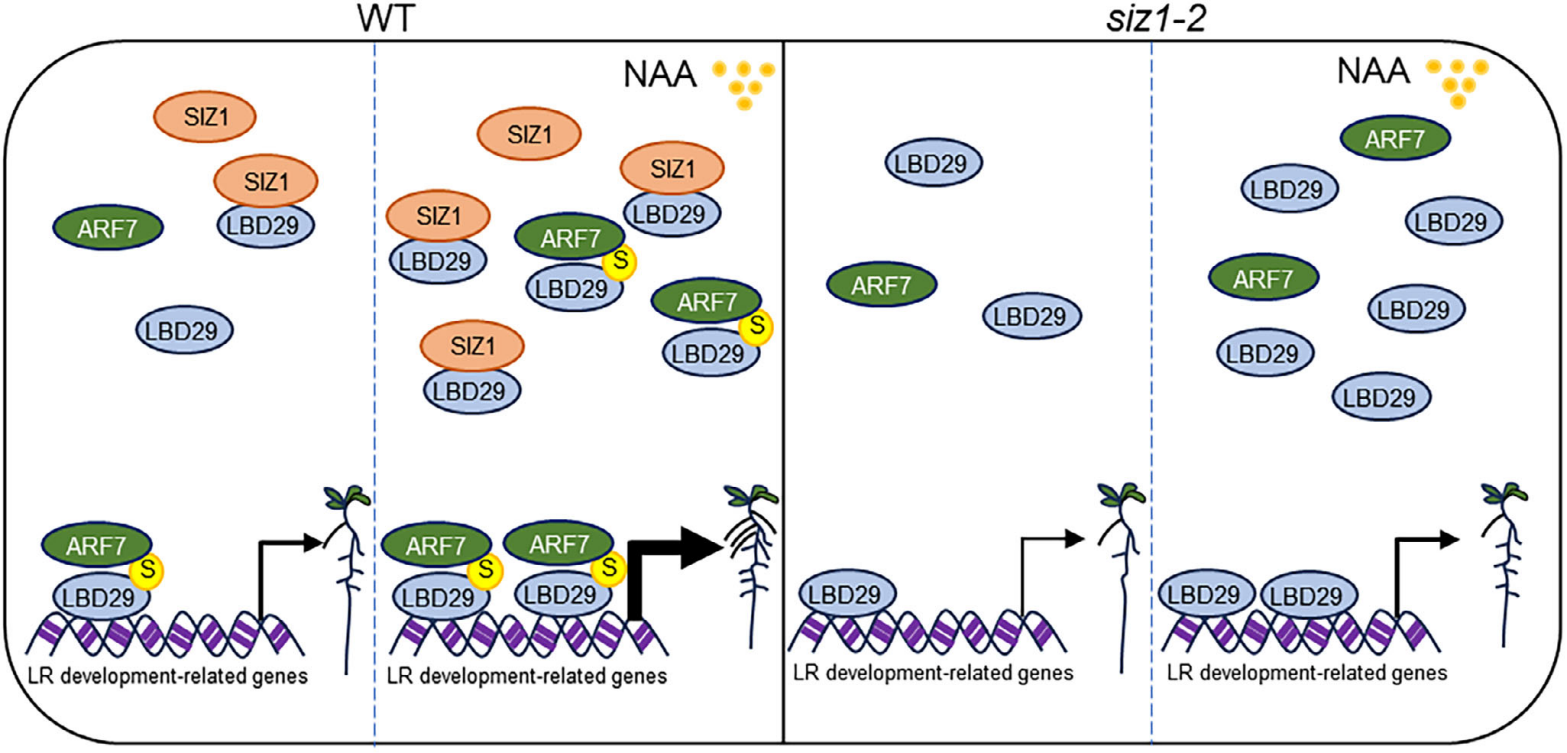
A proposed model of SIZ1‐mediated SUMOylation of LBD29 regulating lateral root formation. In WT, LBD29 interacts with SIZ1 and is modified by SUMO1. Subsequently, SIZ1‐mediated SUMOylation of LBD29 enhances its interaction with ARF7, facilitating transcriptional activation of LR‐promoting genes. As auxin levels increase, activated ARF7/19 promotes the expression of *LBD29* and *SIZ1*; most of the LBD29 is modified by SUMO1, amplifying the regulation of auxin signals on lateral roots (left). In *siz1‐2*, LBD29 cannot interact with ARF7. With the accumulation of auxin, *LBD29* is induced to be expressed, but LBD29 cannot be modified by SIZ1, resulting in low transcriptional activity (right).

Here, we show that auxin activates ARF7/19 and promotes *SIZ1* expression and SIZ1‐mediated SUMOylation, thereby reshaping the interactome of auxin‐responsive proteins. Such large‐scale modulation of the protein network highlights the dynamic and adaptive nature of auxin signaling in response to developmental and environmental cues. Notably, we identified LBD29 as a SUMOylation target that is critical for LR formation. SUMOylation enhanced the interaction between LBD29 and ARF7, stabilizing a transcriptional activation complex that drives the expression of downstream genes, such as *EXP14*, during LR development (Figure 7). This mechanism provides a direct molecular explanation for the reduced auxin responsiveness and impaired LRE observed in *siz1‐2* mutants.

The *siz1* mutants exhibit a distinct phenotype characterized by an increased number of LRP but a decrease in LRE, which suggests that SIZ1 regulates the transition from LRP to emerged LRs. Intriguingly, this phenotype was also observed in some key auxin‐responsive transcription factors such as the *arf7* mutant and the *lbd29* mutant, as well as the *ERF13* overexpression [34]. This parallel strongly implies that SIZ1‐mediated SUMOylation could act as a regulatory hub, integrating diverse signals to control the priming and execution of LRE.

MMS21 SUMOylates IAA17, stabilizing this repressor to dampen the auxin response and regulate primary root elongation [41]. In contrast, SIZ1 SUMOylates LBD29 and promotes its interaction with ARF7 to activate gene expression during LR development. We demonstrated that two distinct SUMO E3 ligases control auxin signaling by modifying key components in opposing ways. This revealed that SUMOylation is not a uniform modifier; it can either reinforce repression or enhance activation depending on the specific E3 ligase and substrate involved. Our findings, together with research on MMS21, establish SUMOylation as a new and critical regulatory layer that modulates auxin response.

ARF7 serves as a critical regulator in LR development, and its dual function as both a substrate and a “reader” of SUMOylation reveals a sophisticated mechanism for fine‐tuning downstream responses under varying environmental conditions. Under normal conditions, ARF7 is not SUMOylated but possesses a functional SIM that enables it to act as a reader of SUMOylation signals. Specifically, ARF7 is recruited by SUMOylated LBD29 via SIM, forming a transcriptional activation complex that promotes the expression of genes required for LR development.

Conversely, under drought stress, ARF7 becomes a substrate for SUMOylation via specific E3 ligases (other than SIZ1). SUMOylated ARF7 enhances its interaction with IAA3, a transcriptional repressor, which in turn inhibits ARF7's transcriptional activity [38]. This shift from reader to substrate status effectively converts ARF7 from an activator to a repressed complex, suppressing LR development as an adaptive‐stress response. These data also suggest that the SIM of ARF7 is an interaction motif and a dynamic regulatory element whose function depends on the SUMOylation status of both ARF7 and its interaction partners. In conclusion, ARF7 exemplifies how a single transcription factor can achieve functional plasticity by alternately serving as a SUMOylation reader under normal conditions and as a substrate under stress. This dual role enables precise environmental control over root architecture, ensuring developmental plasticity while maintaining stress‐adaptation capacity. The interplay between the ARF7 SIM and SUMOylation status creates a sophisticated regulatory node that integrates developmental and stress signals. It would be interesting to investigate the role of the ARF7 SIM in the development of LR.

Collectively, our findings establish a mechanistic framework in which SIZ1‐mediated SUMOylation is integrated with ARF7/LBD29 transcriptional networks to orchestrate LR development. This study highlights the significance of PTM in extending the versatility of auxin signaling and provides new insights into how plants achieve developmental plasticity through multilayered regulation. Future studies should explore the interaction between SIZ1‐mediated SUMOylation and other hormonal and stress signaling pathways and whether dynamic SUMOylation contributes to adaptive responses under fluctuating environmental conditions. Such investigations will deepen our understanding of how PTM‐mediated signaling flexibility underpins plant growth, resilience, and adaptation.

## 4 Materials and Methods

### 4.1 Plant Materials and Growth Conditions

All plant materials used in this study are in the Arabidopsis (*A. thaliana*) Columbia‐0 (Col‐0) background. The *siz1‐2* (SALK_065397) [61], *siz1‐3* (SALK_034008) [57]*, ProSIZ1:SIZ1‐GFP/siz1‐2#*9 [44], *ProSIZ1:SIZ1‐GFP/siz1‐2#10* ^44^, *lbd29* (Salk_071133) [62], *lbd16* (Salk_040739) *lbd18* (Salk_038125) *lbd29* ^33^, and *arf7‐1* (Salk_040394) *arf19‐*1 [63] were described previously. The *Pro35S:LBD29‐6Gly‐FLAG* was constructed by introducing the *LBD29* coding sequence (CDS) into the *PK7WG2* binary vector and subjected to transform WT plants. The *Pro35S:SIZ1‐GFP* was constructed by introducing the *SIZ1* coding sequence (CDS) into the *pSuper1300‐GFP* binary vector and subjected to transform WT plants. The 3,000 regions upstream of *LBD29* start codons were amplified and linked to the vector *pQ‐GG* with GFP to obtain *ProLBD29:LBD29^WT^‐GFP/lbd29#1*, *ProLBD29:LBD29^WT^‐GFP/lbd29#2*, *ProLBD29:LBD29^K21/24R^‐GFP/lbd29#5*, *ProLBD29:LBD29^K21/24R^‐GFP/lbd29#6*. The *Pro35S:LBD29‐6Gly‐FLAG/siz1‐2* line was generated by crossing *Pro35S:LBD29‐6Gly‐FLAG* and *siz1‐2* plants. The *lbd29/siz1‐2* line was generated by crossing *lbd29* and *siz1‐2* plants. The plants were grown on 1/2 Murashige and Skoog (1/2 MS) medium with 0.8% agar (w/v), and then incubated at 4°C for 2 d, and were finally transferred to a plant growth chamber with a 16‐h photoperiod at a constant 22°C for 8 d. A 10 µM/50 nM NAA (Sigma, N0640) and a 0.5 µM IAA (Sigma, I3750) solution were employed for treating the seedlings.

### 4.2 RNA Sequencing Analysis

The RNA submitted for RNA‐seq analysis was isolated from the roots of 8‐d‐old Col‐0 and *siz1‐2 Arabidopsi*s seedlings using an RNeasy Mini Kit (TIANGEN, DP419, PK145). The extracted RNA was sent to Oebiotech for the RNA‐SEQ project. The data analysis platform provided by Oebiotech was used for data analysis. Sequencing data are available in the Gene Expression Omnibus (GEO) database under accession number: GSE271343.

### 4.3 Yeast One‐Hybrid Assay

For the Y1H assay, the coding sequences of proteins were amplified and cloned into *pGADT7‐DEST* (AD) by *Nde* I and *Nco* I, and the sequences of the target promoters (2400 bp upstream of ATG) were introduced to *pHIS2.1* by *EcoR* I and *Sma* I, respectively. The built‐up vectors were then transformed into Y187 yeast cells (Clontech). The yeast transformation method process was basically consistent with the Y2H assay, except SD/‐Leu/‐Trp/‐His (SD‐LWH) culture medium was used in interactions. Primers used for Y1H assays are listed in Table S3.

### 4.4 Yeast Two‐Hybrid Assay

To construct the vectors required for the Y2H assay using homologous recombination. The *pGADT7* (Clontech) or *pGBKT7* (Clontech) was linearized using *Nde* I and *EcoR* I restriction enzymes. Subsequently, the full‐length sequences, including ARF7 and SIZ1, were individually cloned into the multiple cloning sites of the *pGADT7* vector. Additionally, the full‐length sequences of LBD29^WT^, LBD29^K21/24R^, LBD29^SUMO1^were separately inserted into the *pGBKT7* vector. The built‐up vectors were then transformed into Gold Yeast Cells (Clontech), and yeast cells were grown on SD/‐Leu/‐Trp and SD/‐Leu/‐Trp/‐His or SD/‐Leu/‐Trp/‐His/‐Ade medium but supplemented with AbA (Aureobasidin A) and X‐α‐gal (5‐bromo‐4‐chloro‐3‐indolyl‐b‐*d*‐galactopyranoside). The autoactivation was inhibited by the addition of 3‐amino‐1,2,4‐triazole (3‐AT). Primers used for Y2H assays are listed in Table S3.

### 4.5 In Vitro Pull‐Down

The coding sequences of SIZ1 and LBD29 were cloned into pET‐30a and pGEX‐4T‐1 vectors to generate recombinant His‐SIZ1 and GST‐LBD29, respectively. These recombinant proteins were expressed in *Escherichia coli* BL21 cells (DE3) and purified using Ni NTA Beads (Smart‐Lifesciences Biotech) and Glutathione Beads (Smart‐Lifesciences Biotech) according to corresponding to their respective manuals. GST‐LBD29 or GST was immobilized on Glutathione Beads and then incubated with His‐SIZ1 at 4°C for 4 h. Subsequently, the Glutathione Beads were washed five times with 1 × PBS buffer. The proteins bound to the beads were analyzed by Western blot. His‐tag and GST‐tag were detected by anti‐His antibody (HT501, TransGen Biotech) and anti‐GST antibody (HT601, TransGen Biotech), respectively.

### 4.6 Co‐Immunoprecipitation Assay

For the co‐IP assay, the CDS of SIZ1, LBD29^WT^, LBD29^K21/24R^, LBD29^SUMO1^, ARF7^WT^, ARF7^2SIM^ were cloned into the pSuper1300‐GFP [64] and pSuper1300‐MYC [64] vectors, respectively, to generate SIZ1‐GFP, LBD29‐MYC, LBD29‐GFP, LBD29^K21/24R^‐GFP, LBD29^SUMO1^‐GFP, ARF7‐MYC, ARF7‐GFP, ARF7^2SIM^‐GFP. Protoplasts were prepared as previously described [65]. SIZ1‐GFP and LBD29‐MYC were co‐expressed in Arabidopsis protoplasts, with SIZ1‐GFP or LBD29‐MYC being individually expressed as negative controls. Total proteins were extracted using NP‐40 lysis buffer (Beyotime Biotech) supplemented with 1 mM phenylmethylsulfonyl fluoride (PMSF). Then the extracts were incubated with GFP‐Trap agarose beads (Chromo Tek, Cat# ytma) at 4°C for 2 h. After washing three times with dilution buffer (10 mM Tris‐HCl, pH 7.5, 150 mM NaCl, 0.5 mM EDTA), the bound proteins were eluted with 2×SDS loading buffer and detected by Western blotting. The YFP‐tag and MYC‐tag were detected using anti‐GFP antibody (HT801‐02, TransGen Biotech) and anti‐MYC antibody (AE010, ABclonal), respectively.

### 4.7 Luciferase Complementation Imaging Assay

For the LCI assay, the coding sequence of SIZ1 was cloned into the *JW771* vector to generate the SIZ1‐nLUC fusion protein. Similarly, the coding sequences of LBD29 and LBD29‐N/C were cloned into the *JW772* vector to generate LBD29‐cLUC and LBD29‐N/C‐cLUC fusion proteins, respectively. For the SIZ1‐LBD29 interaction, the SIZ1‐nLUC + LBD29‐cLUC, SIZ1‐nLUC + cLUC, LBD29‐cLUC + nLUC combinations were co‐transfected into *N. Benthamiana* leaves. After growth in darkness for 12 h, the infiltrated *N. benthamiana* plants were transferred to light and grown at 26°C for 48 h. The leaves were sprayed with D‐luciferin potassium salt (GOLDBIO, Lot# 014130LUCK) for 10 min, and the luminescence was observed with a Tanon‐5200 Multi imaging system (Tanon, Shanghai, China) [65]. Primers used for LCI assays are listed in Table S3.

### 4.8 In Vivo SUMOylation Assay

Total proteins were extracted from Col‐0 and *siz1‐2* seedlings treated with 10 µM NAA for 0, 12, and 24 h using ice‐cold lysis buffer (50 mM Tris‐HCl [pH 8.0], 120 mM NaCl, 0.2 mM sodium orthovanadate, 100 mM NaF, 10% [v/v] glycerol, 0.2% [v/v] Triton X‐100, 5 mM DTT, 1× protease inhibitor cocktail, 5 mM N‐ethylmaleimide). Proteins were resolved by SDS‐PAGE. Immunoblotting was performed with anti‐AtSUMO1 antibody (1:2000 dilution; PHYTOAB, PHY1049A). Protein signals were visualized using the ECL Plus chemiluminescence detection system (Epizyme, Q101L).

The in vivo SUMOylation detection was conducted using Col‐0*/siz1‐2 Arabidopsis* protoplasts transiently expressing LBD29^WT^‐MYC (50 µg) or LBD29^K21/24R^‐MYC (50 µg) and FLAG‐SUMO1‐GG/FLAG‐SUMO1‐ΔGG (80 µg). Proteins were extracted in the presence of N‐ethylmaleimide (NEM) (E3876, Sigma) and a protease inhibitor cocktail (Roche, 04693159001). After immunoprecipitation (IP) using anti‐MYC antibody, the beads were washed, and proteins were eluted with 2×SDS loading buffer. Western blotting was performed using anti‐MYC (AE010, ABclonal) and anti‐FLAG (AE169PM, ABclonal) antibodies.

The in vivo SUMOylation detection was conducted using *N. benthamiana* leaves transiently expressing LBD29‐MYC and FLAG‐SUMO1‐GG. After growth in darkness for 12 h, the infiltrated *N. benthamiana* plants were transferred to light and grown at 26°C for 48 h. Then the leaves were treated with 10 µM NAA for 12 h and 24 h, respectively. Total proteins were extracted using lysis buffer (50 mM Tris‐HCl pH 8.0, 120 mM NaCl, 0.2 mM phosphatase inhibitor, 100 mM NaF, 10% glycerol, 0.2% Triton X‐100, 5 mM DTT) supplemented with protease inhibitor cocktail and 5 mM NEM. The extracted proteins were incubated with anti‐MYC magnetic beads at 4°C overnight with rotation. After washing, the beads were collected and resuspended in 2×SDS loading buffer, followed by boiling at 95°C for 5 min. Western blotting was performed using anti‐MYC antibody for detecting LBD29‐MYC and anti‐SUMO1 (PHY3847S, PhytoAB) antibody for detecting SUMOylation.

### 4.9 In Vitro SUMOylation Assay

The SUMOylation assay in *Escherichia coli* was carried out according to the procedures previously described [66]. The CDS of LBD29 was cloned into the *pCDFDuet‐*1 [67] vector with a C‐terminal Flag fusion. The constructed plasmids were transformed for protein expression in the *E. coli* BL21(DE3) carrying *pET28‐SAE1a‐His6‐SAE2* with *pACYCDuet‐1‐MYC‐SUMO1GG* or *pACYCDuet‐1‐His6‐SCE1‐MYC‐SUMO1GG*. After being cultured in LB medium with 1 mM IPTG at 37°C for 16 h, the cells were harvested for immunoblotting using an anti‐Flag antibody. This assay uses primers listed in Table S3.

### 4.10 Cell‐Free Protein Degradation Assay

Cell‐free experiments were performed as previously described [68]. In brief, 14‐d‐old WT seedlings treated with ^1/2^MS medium and 10 µM NAA for 4 h, respectively, were ground into powder in liquid nitrogen, followed by transfer of 1.2 g of powder to a precooled 10.0 mL centrifuge tube and the addition of 1.2 mL of nondenaturing protein extraction buffer [10 mM NaCl, 25 mM tris‐HCl (pH 7.5), 4 mM phenylmethylsulfonyl fluoride, 10 mM adenosine 5′‐triphosphate, 5 mM DTT, and 10 mM MgCl_2_] and incubation on ice for 30 min with intermittent vertexing every 10 min. After centrifugation at 4°C at 14 000 *g* for 15 min, 1.2 mL of supernatant was collected and divided into three 2.0 mL centrifuge tubes. Then, 80 µl of recombinant HIS‐SIZ1 was added to one of the three centrifuge tubes and incubated at 22°C for 0, 5, 15, or 30 min. At each time point, 100 µL of the reaction solution was taken out, and 25 µL of 5× loading buffer was added to stop the reaction. Immunoblotting was used to detect.

### 4.11 Protein Degradation Assay

To verify the effects of auxin on SIZ1 protein levels, 10‐d‐old *35S::SIZ1‐GFP* seedlings were treated with 150 µM CHX and 150 µM CHX+10 µM NAA for 3, 6, and 12 h, respectively, and were ground into powder in liquid nitrogen, followed by transfer of 1.2 g of powder to a precooled 10.0 mL centrifuge tube and the addition of 1.2 mL of nondenaturing protein extraction buffer [1%NP‐40, 1 mM PMSF] and incubation on ice for 30 min with intermittent vertexing every 10 min. After centrifugation at 4°C at 14 000 *g* for 15 min, 1.2 mL of supernatant was collected and divided into three 2.0 mL centrifuge tubes. Immunoblotting was used to detect.

### 4.12 Protein IP, LC‐MS/MS, and Data Analysis

Protein Immunoprecipitation (IP): 3 g of roots from 10‐day‐old *Pro35S:SIZ1‐GFP* and *Pro35S: GFP* seedlings were ground into a fine powder in liquid nitrogen. The powder was immediately transferred to a pre‐chilled 50 mL centrifuge tube and resuspended in 3 mL of ice‐cold lysis buffer [1% (v/v) Triton X‐100, 150 mM NaCl, 50 mM Tris‐HCl (pH 7.5), 5 mM EDTA, supplemented with protease inhibitor cocktail tablets]. The samples were lysed on ice for 30 min, followed by centrifugation at 14 000 *g* for 15 min at 4°C. The supernatant was transferred to a new 50 mL tube, and 30 µL of GFP‐Trap magnetic beads (Chromo Tek, Cat# ytma) were added. The mixture was incubated at 4°C with gentle rotation for 4 h. The beads were collected and washed three times with lysis buffer.

For liquid chromatography–tandem MS (LC‐MS/MS), the IP beads were sent to Shanghai Bioprofile (Shanghai, China) for analysis. Protein was extracted from tissue samples using SDT lysis buffer (4% SDS, 100 mM DTT, 100 mM Tris‐HCl pH 8.0). Samples were boiled for 3 min and further ultrasonicated. Undissolved cellular debris was removed by centrifugation at 16 000 *g* for 15 min. The supernatant was collected and quantified with a BCA Protein Assay Kit (BeyoTime, China). LC‐MS/MS was performed on an Orbitrap Astral mass spectrometer coupled with a Vanquish Neo UHPLC system (Thermo Fisher Scientific).

### 4.13 Chromatin Immunoprecipitation‐qPCR Assay

3 g roots were obtained from 8‐d‐old plant grown seedlings *Pro35S:LBD29‐6Gly‐FLAG×siz1‐2*, *Pro35S:LBD29‐6Gly‐FLAG* or *ProLBD29:LBD29^WT^‐GFP/lbd29*, *ProLBD29:LBD29^K21/24R^‐GFP/lbd29*. Protoplasts were prepared as previously described [69]. The roots were fixed in formaldehyde, chromatin was clipped to an average length of 200–500 bp and then immunoprecipitated with anti‐FLAG/anti‐GFP. After purification by reverse crosslinking, immunoprecipitation, and input DNA were quantitatively analyzed by RT‐PCR. ChIP enrichment was assessed by comparing the threshold period of ChIP samples with their own input. The resulting data are presented as fold change relative to Actin. qPCR using primers listed in Table S3.

### 4.14 Transient Expression

The CDS of *LBD29^WT^*, *LBD29^K21/24R^*, *ARF7*, and *ARF7^2SIM^* were amplified, and the resulting sequences were introduced into *pBI221* to place them under the control of the *CaMV35S* promoter. The *EXP14* promoter sequences were amplified and introduced into the *pGreenII0800‐LUC* reporter vector. Both recombinant plasmids were then transferred into Col‐0/*siz1‐2 Arabidopsis* protoplasts. Firefly luciferase and Renilla luciferase activities were measured using the Dual‐Luciferase Reporter Assay System. LUC activity was normalized against Renilla luciferase activity.

### 4.15 Quantitative Real‐Time Polymerase Chain Reaction

8‐d‐old Col‐0 seedlings were treated with 10 µm NAA for 1, 3, 6, 12, and 24 h, and ∼0.1 g of root tissue was collected for total RNA extraction. 8‐d‐old Col‐0, *arf7*, *arf7/19*, and *lbd16/18/29* seedlings were collected for total RNA extraction. For qRT‐PCR, RNA was isolated from roots using a RNeasy Plant Mini Kit (TIANGEN, DP419, PK145) following the manufacturer's protocol. The RNA was then reverse‐transcribed into cDNA using HiScript III RT SuperMix for qPCR (+gDNA wiper) (Vazyme, #R323). Quantitative PCR (qPCR) was performed on a MyiQ Real‐time PCR Detection System (Bio‐Rad, Hercules, CA, USA) using the ChamQ SYBR Color qPCR Master Mix (Q411, Vazyme, Nanjing, China). *ACTIN2* (At3g18780) was used as an internal control. Three biological replicates were performed, and each biological replicate was represented by three technical replicates. Primers used for qRT‐PCR are listed in Table S3.

### 4.16 Light Microscopy and Confocal Imaging

To visualize LR, 8‐day‐old seedlings underwent treatment following a previously described procedure [62]. Observation and imaging were conducted using an Olympus BX53 microscope equipped with a DP72 camera and cellSens Standard 1.6 software.

For PI staining, 8‐d‐old *Arabidopsis* seedlings were incubated with 1 mg mL^−1^ PI solution (Sangon Biotech) for 1 min. Subsequently, the treated seedlings were placed on a microscope slide for observation and imaging using a Zeiss LSM880 confocal microscope. During imaging, GFP was excited at a wavelength of 488 nm and detected within a range of 493–594 nm. PI was excited at 488 nm and detected within a range of 600–630 nm.

### 4.17 Accession Numbers

All genes sequence information in this article can be found in *Arabidopsis* Genome Initiative according to accession numbers as follows: *SIZ1* (AT5G60410), *LBD29* (AT3G58190), *ARF7* (AT5G20730), *ARF19* (AT1G19220), *EXP14* (AT5G56320), *SUMO1* (AT4G26840), *LAX3* (AT1G77690), *STOP1* (AT1G34370).

### 4.18 Statistical Analysis

Statistical analyses were performed using SPSS software (version 21, IBM) with Student's *t*‐tests and one‐way ANOVA with Tukey's multiple comparison test to determine significance. For Student's *t*‐tests, a *p*‐value < 0.05 was considered statistically significant, with asterisks indicating different significance levels (^*^ *p* < 0.05, ^**^ *p* < 0.01, and ^***^ *p* < 0.001). Similarly, for one‐way ANOVA with Tukey's multiple comparison test, *P*‐values < 0.05 were regarded as statistically significant. All data were presented as means and standard error or standard deviation. Statistical details can be found in the figure legends, including biological repeats.

## Author Contributions

X.K. and Z.D. designed the research. J.S., Q.Y., Y.C., and L.Z performed the research. J.S., Q.Y., Z.D., X.K., and X. Y. analyzed data. J.S., Q.Y., X.K., and Z.D. wrote the article.

## Conflicts of Interest

The authors declare no conflicts of interest.

## Supporting information




**Supporting File 1**: advs76633‐sup‐0001‐SuppMat.pdf.

**Supporting File 2**: advs76633‐sup‐0002‐TableS1.xlsx.

**Supporting File 3**: advs76633‐sup‐0003‐TableS2.xlsx.

**Supporting File 4**: advs76633‐sup‐0004‐TableS3.xlsx.

## Data Availability

The data that support the findings of this study are available on request from the corresponding author. The data are not publicly available due to privacy or ethical restrictions.
