# Supplementary material for: SIZ1‐Mediated SUMOylation of LBD29 Recruits ARF7 to Fine‐Tune Auxin Signaling in Lateral Root Development
Source: Adv Sci (Weinh). 2026 Jul 17;13(56):e76633. doi: 10.1002/advs.76633 (PMC13379264; doi:10.1002/advs.76633)
Supplement: Supplementary file 1 [file ADVS-13-e76633-s001.pdf]

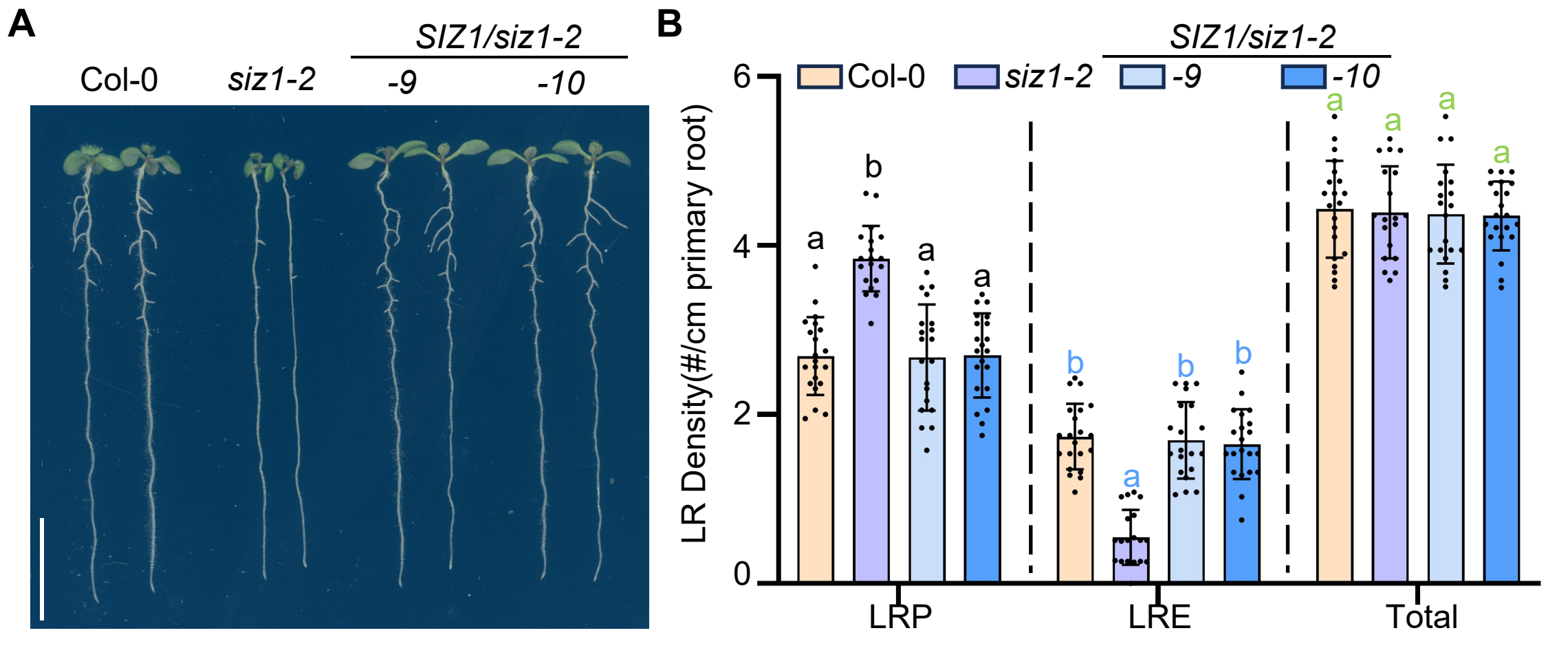

Figure S1 LR phenotype of the *siz1-2* complementation line

(A) LR phenotypes were examined in 8-d-old seedlings, including Col-0, *siz1-2*, *SIZ1/siz1-2* (*ProSIZ1:SIZ1/siz1-2*) *#9* and *SIZ1/siz1-2#10*. Scale bar, 1 cm.

(B) LR density of Col-0, *siz1-2*, *SIZ1/siz1-2#9* and *SIZ1/siz1-2#10*. Three independent biological replicates produced similar results, and at least 19 seedlings per line were used for statistical analysis. Different lowercase letters indicate significant differences by one-way ANOVA, followed by Tukey's multiple comparison test ($P < 0.05$). Data are indicated as means ± SD ($n \geq 19$).

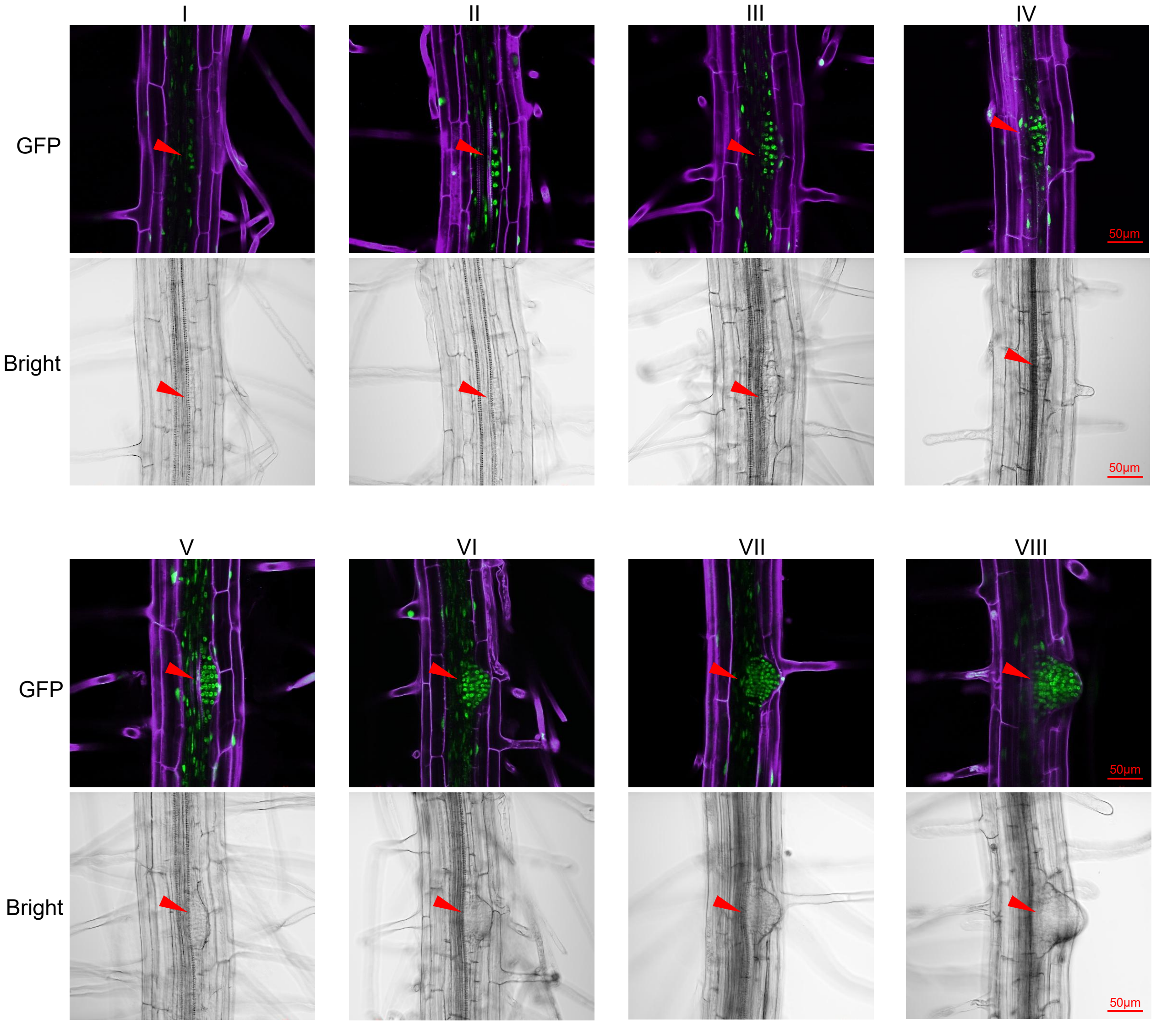

Figure S2 *SIZ1* is expressed at stages I to VIII of LR development

Fluorescence images of the LRP of 8-d-old *ProSIZ1:SIZ1-GFP/siz1-2* seedlings. In all fluorescent images, we replaced red (propidium iodide signal) with magenta. Bar, 100 μm.

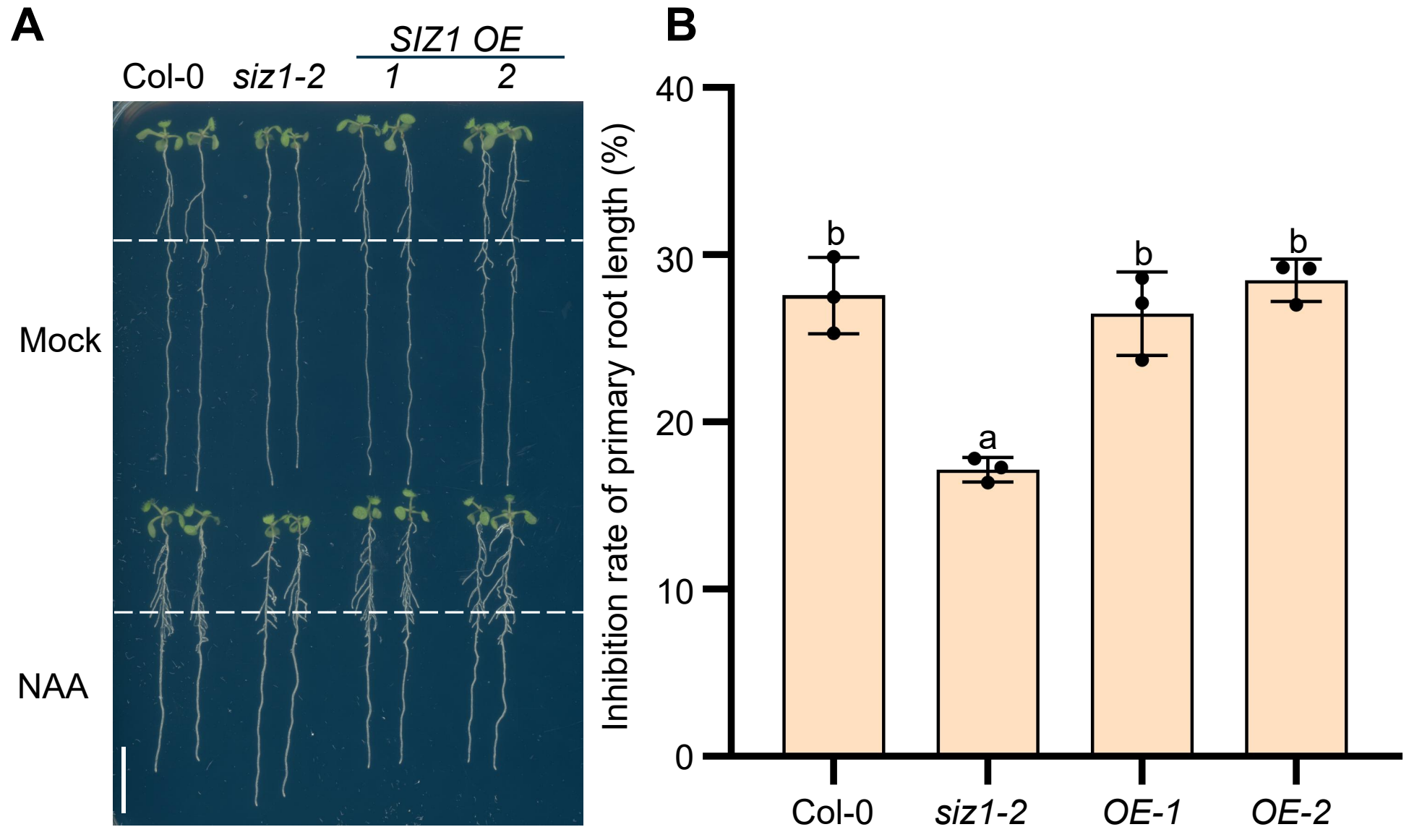

Figure S3 SIZ1 positively regulates auxin-mediated primary root elongation

(A) LR phenotypes were assessed in 10-d-old seedlings of Col-0, *siz1-2* and *SIZ1*-overexpressing lines. Scale bar, 1 cm. Col-0, *siz1-2* and *SIZ1*-overexpressing seedlings grown on $^{1/2}$MS medium for 5 days were transferred to $^{1/2}$MS medium supplemented with 100 nM NAA and grown for an additional 5 days.
(B) The degree of inhibition of the main root length in (A). Three independent biological replicate experiments all produced similar results, and at least 12 seedlings of each strain were used for statistical analysis. Each point represents one repetition, and different lowercase letters indicate significant differences obtained through one-way ANOVA, followed by Tukey's multiple comparison test ($P < 0.05$). Data are indicated as means ± SD (n = 3).

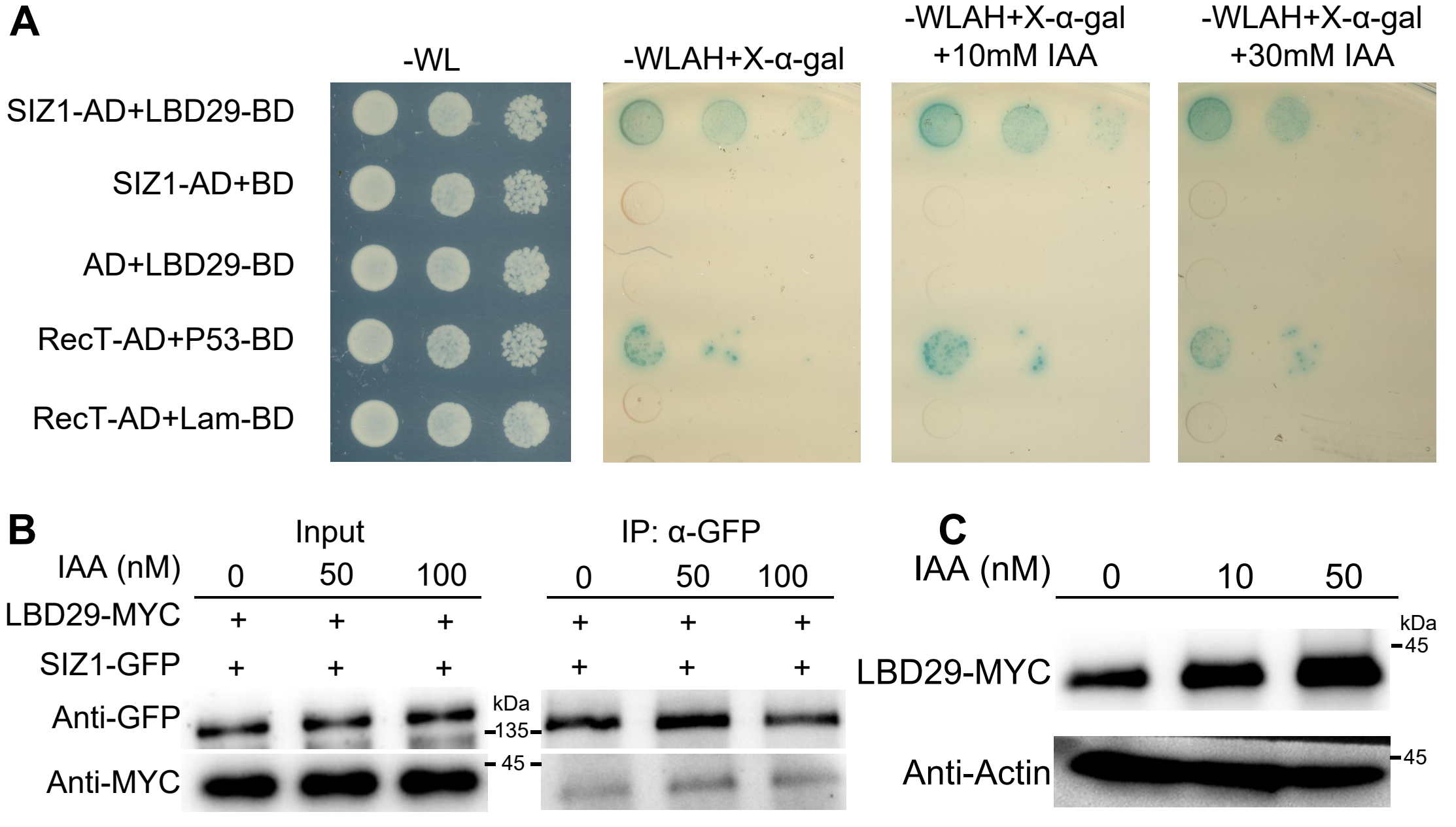

Figure S4 Auxin slightly promotes the interaction between SIZ1 and LBD29, but induces LBD29 protein accumulation

(A) Y2H assay to detect the effect of auxin on the interaction between LBD29 and SIZ1. Yeast cells co-transformed *pGADT7-SIZ1* and *pGBKT7-LBD29* were dropped onto SD-WL, SD-WLAH + X-α-gal, SD-WLAH + X-α-gal +10 mM IAA/30 mM IAA medium. The negative control and positive control were transformed with commercial plasmids. AD, activation domain; BD, DNA binding domain.
(B) co-IP assays were performed in *Arabidopsis* protoplasts to investigate the effect of auxin on the interaction between LBD29 and SIZ1. Protoplasts were isolated from leaves of Col-0 and co-transformed with *SIZ1-GFP* and *LBD29-MYC* constructs. After overnight incubation, the protoplasts were treated with 50 or 100 nM IAA for 4 h. Protein extracts were then subjected to western blot analysis.
(C) A transient transformation assay in protoplasts was performed to verify that auxin promotes the accumulation of LBD29 protein. Protoplasts were isolated from leaves of Col-0 and transformed *LBD29-MYC* constructs. After overnight incubation, the protoplasts were treated with 10 or 50 nM IAA for 4 h. Protein extracts were then subjected to western blot analysis.

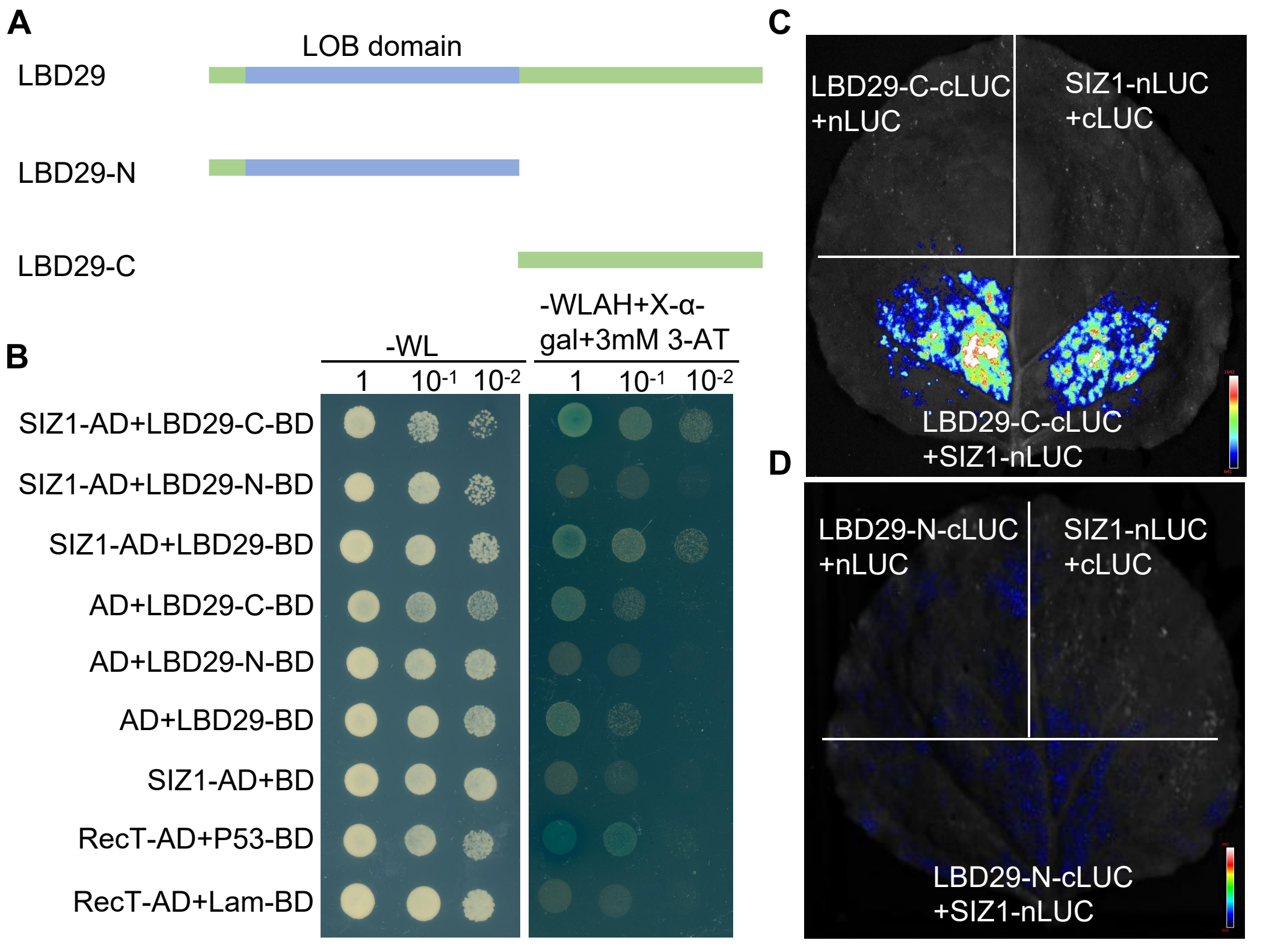

Figure S5 Physical interactions of SIZ1 protein with LBD29-N/C

(A) Schematic diagram of LBD29 protein structure.
(B) Y2H assays showing the LBD29-C/N interacts with SIZ1. Yeast cells were grown on SD-WL and SD-WLAH + X-α-gal + 3 mM 3-AT medium. Yeast cells co-transformed with *pGADT7-LBD29-C/N* and *pGBKT7-SIZ1* were dropped onto SD-WL and SD-WLAH + X-α-gal + 3 mM 3-AT medium.
(C) LCI assay showing that LBD29-C interacts with SIZ1. Three different combinations, including two negative controls, were separately injected into four different areas of the leaf. nLUC/cLUC, N-terminal/C-terminal luciferase.
(D) LCI assay showing that LBD29-N interacts with SIZ1. Three different combinations, including two negative controls, were separately injected into four different areas of the leaf. nLUC/cLUC, N-terminal/C-terminal luciferase.

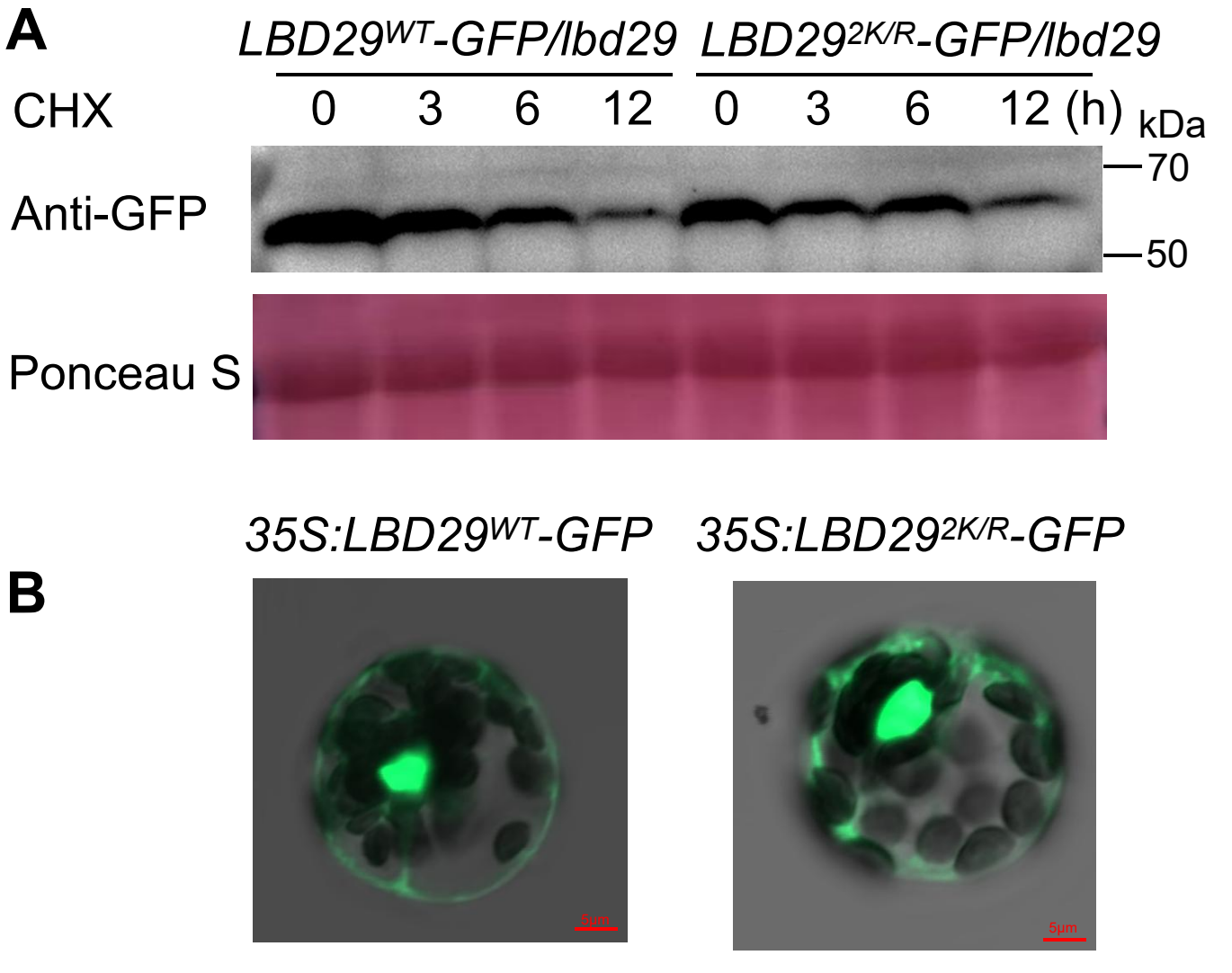

Figure S6 SUMOylation does not affect the protein stability or subcellular localization of LBD29

(A) The SUMOylation modification does not affect the protein stability of LBD29, *LBD29^{WT}-GFP/lbd29* and *LBD29$^{2K/R}$-GFP/lbd29* seedlings were treated with 150 μM CHX for 3, 6, and 12 h, respectively. Protein extracts were then subjected to western blot analysis to examine the protein stability of LBD29^{WT}-GFP and LBD29$^{2K/R}$-GFP.
(B) The SUMOylation modification does not affect the subcellular localization of LBD29. The *35S:LBD29^{WT}-GFP* and *35S:LBD29$^{2K/R}$-GFP* plasmids were separately transformed into protoplasts isolated from Col-0, and the GFP signals were then observed using a confocal laser scanning microscope. Bar, 50 μm.

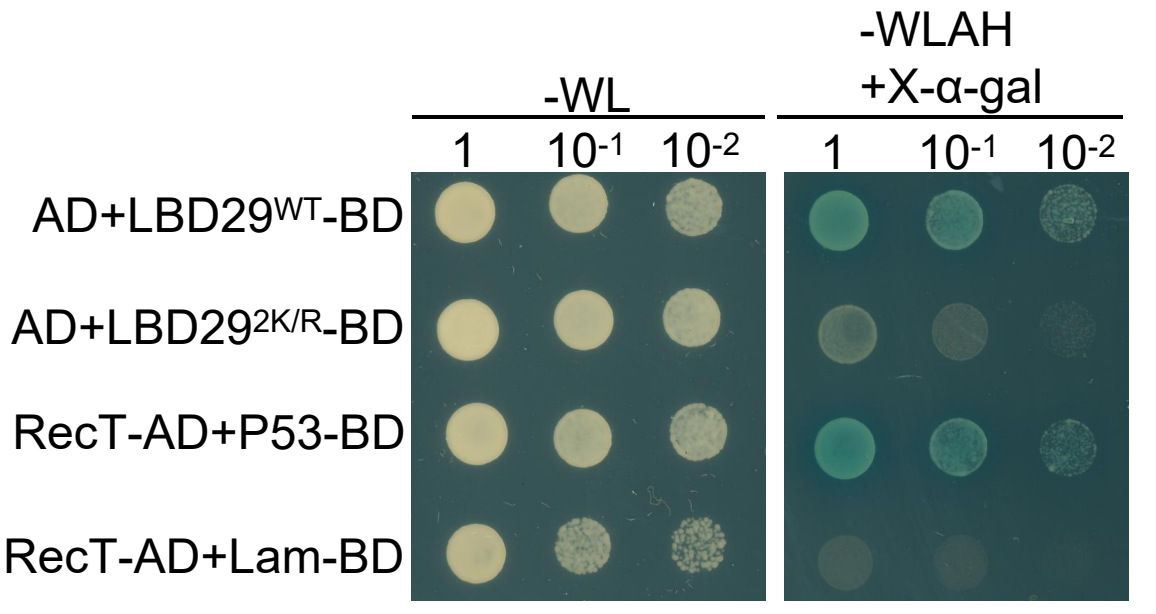

Figure S7 Y2H autoactivation test of LBD29

Interaction of $LBD29^{WT}$ or $LBD29^{2K/R}$ with AD in a Y2H assay. Yeast cells were grown on SD-WL and SD-WLAH + X-α-gal medium. Yeast cells co-transformed with *pGADT7* and *pGBKT7-LBD29*^{WT} or *pGBKT7-LBD29*$^{2K/R}$ were dropped onto SD-WL and SD-WLAH + X-α-gal medium. The negative control and positive control were transformed with commercial plasmids. AD, activation domain; BD, DNA binding domain.

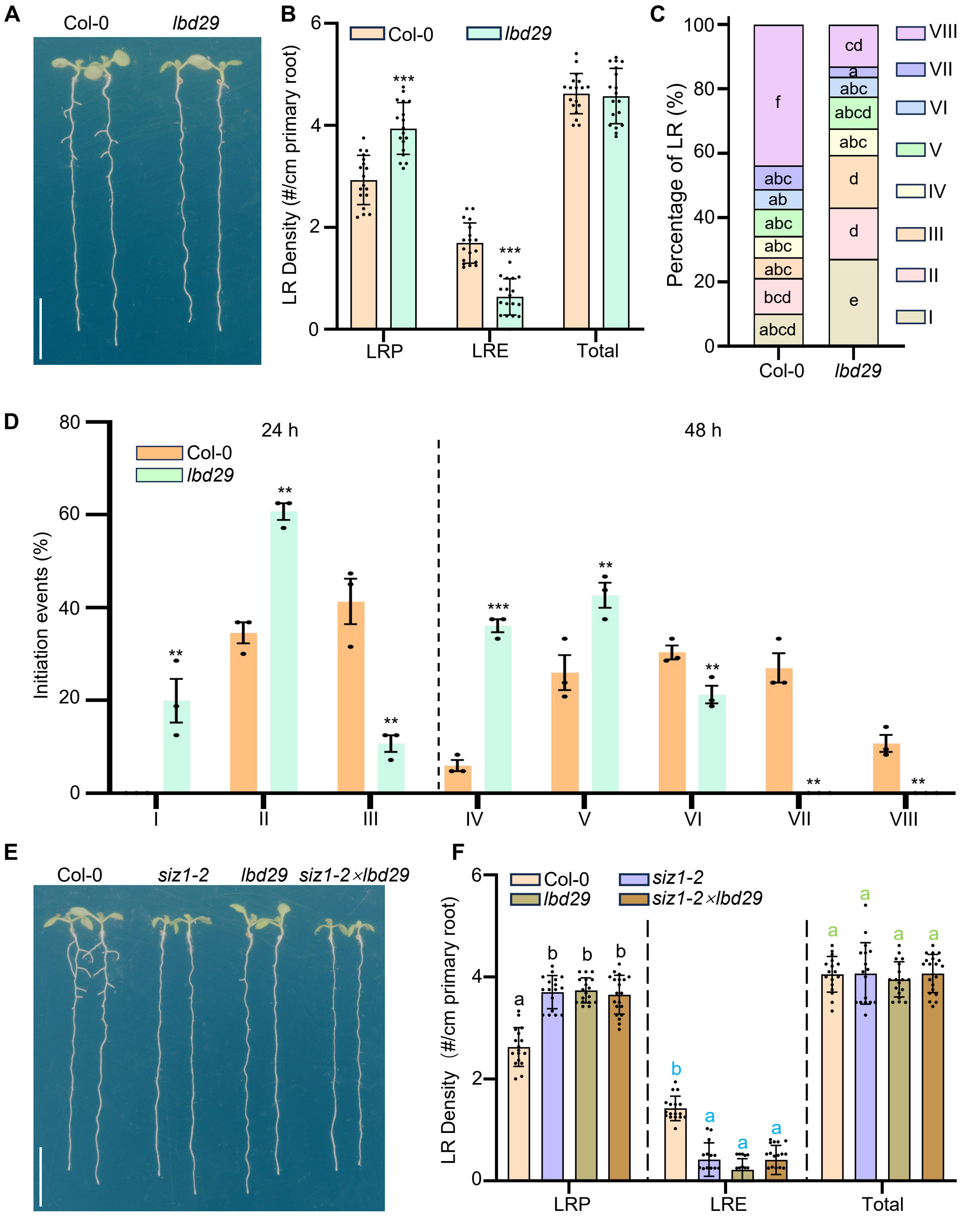
A
Col-0
lbd29
B
LR Density (#/cm primary root)
Col-0
lbd29
***
***
LRP
LRE
Total
C
Percentage of LR (%)
f
abc
ab
abc
abc
abc
bcd
abcd
cd
a
abc
abcd
abc
d
d
e
VIII
VII
VI
V
IV
III
II
I
Col-0
lbd29
D
Initiation events (%)
24 h
48 h
Col-0
lbd29
**
***
I
II
III
IV
V
VI
VII
VIII
E
Col-0
siz1-2
lbd29
siz1-2×lbd29
F
LR Density (#/cm primary root)
Col-0
siz1-2
lbd29
siz1-2×lbd29
LRP
LRE
Total

Figure S8 LR phenotype of the *lbd29*

(A) LR phenotypes were assessed in 8-d-old seedlings of Col-0 and *lbd29*. Scale bar, 1 cm.
(B) LR density of Col-0 and *lbd29*. Three independent biological replicates produced similar results, and at least 18 seedlings per line were used for statistical analysis. All plant materials were compared with the Col-0. ***$P < 0.001$, as determined by a Student's *t* test. Data are indicated as means ± SD ($n \geq 18$).
(C) The proportion of LR at different stages were analyzed in 8-d-old seedlings of WT and *lbd29*. Roman numerals I-VIII represent LR developmental stages. Different letters indicate significant differences of the same developmental stage LR proportion among different plants used one-way ANOVA ($P < 0.05$, $n \geq 17$).
(D) The synchronized initiation of LRP was induced using gravitropic stimulation at the site of root bending in the 3-d-old seedlings of Col-0 and *lbd29* (~30 seedlings were measured in each material). Phenotypic analysis of LRP stage I and stage II, stage III to E was achieved after 24 h and 48 h gravistimulus compared with Col-0, respectively. As determined by a Student's *t* test. **$P < 0.01$, ***$P < 0.001$. Data are indicated as means ± SE ($n = 3$).
(E) LR phenotypes were assessed in 8-d-old seedlings of Col-0, *siz1-2*, *lbd29*, and *siz1-2×lbd29*. Scale bar, 1 cm.
(F) LR density of Col-0, *siz1-2*, *lbd29*, and *siz1-2×lbd29*. Three independent biological replicates produced similar results, and at least 20 seedlings per line were used for statistical analysis. Different lowercase letters indicate significant differences by one-way ANOVA, followed by Tukey's multiple comparison test ($P < 0.05$). Data are indicated as means ± SD ($n \geq 20$).

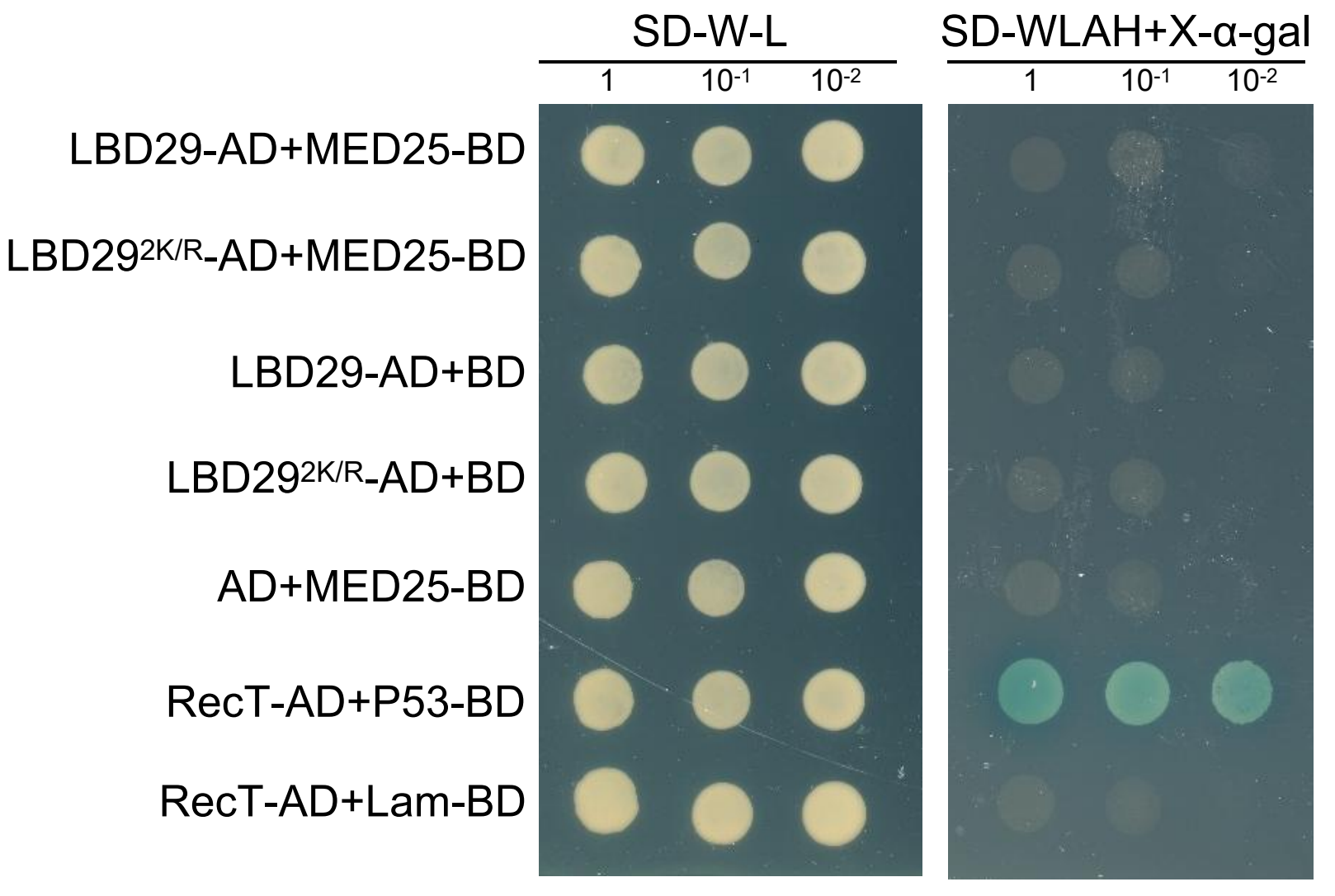

Figure S9 LBD29 does not interact with MED25

Interaction is indicated by the ability of cells to grow on SD/-WL medium and SD/-WLAH + X-α-gal medium for 3 days after plating. The negative control and positive control were transformed with commercial plasmids. AD, activation domain; BD, DNA binding domain.

A

Protein: ARF7

Sample	Intensity
Pro35S:GFP	43013.1
Pro35S:SIZ1-GFP	141942.5

B

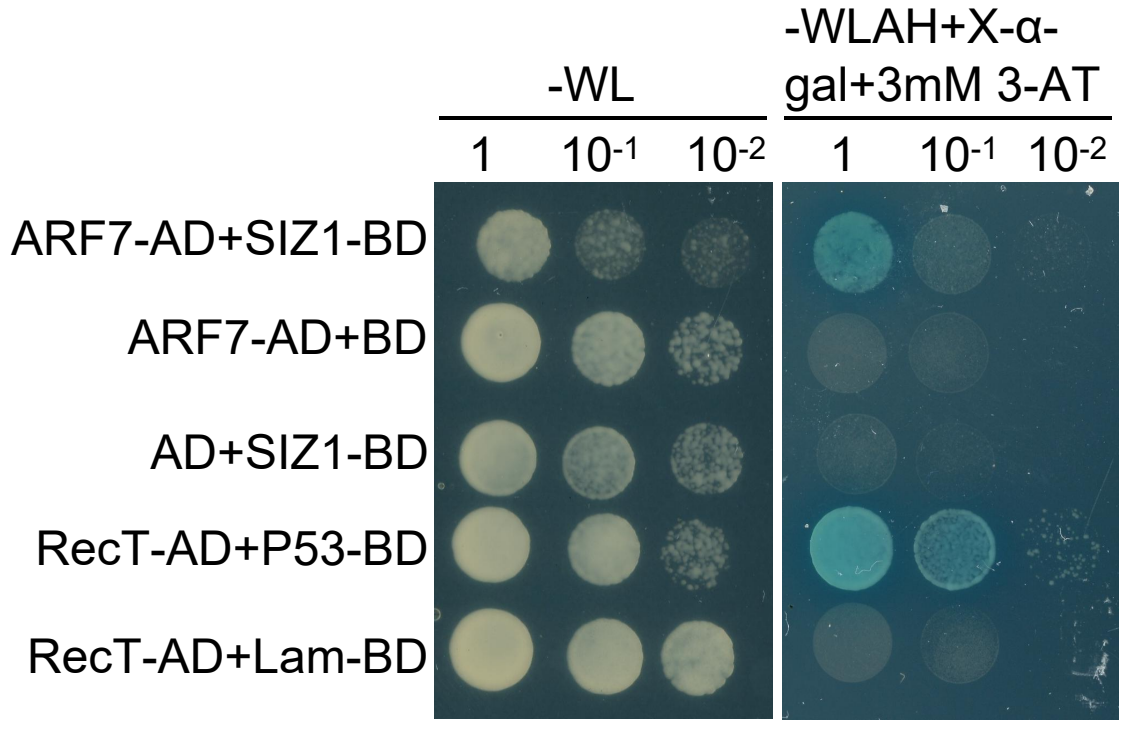

C

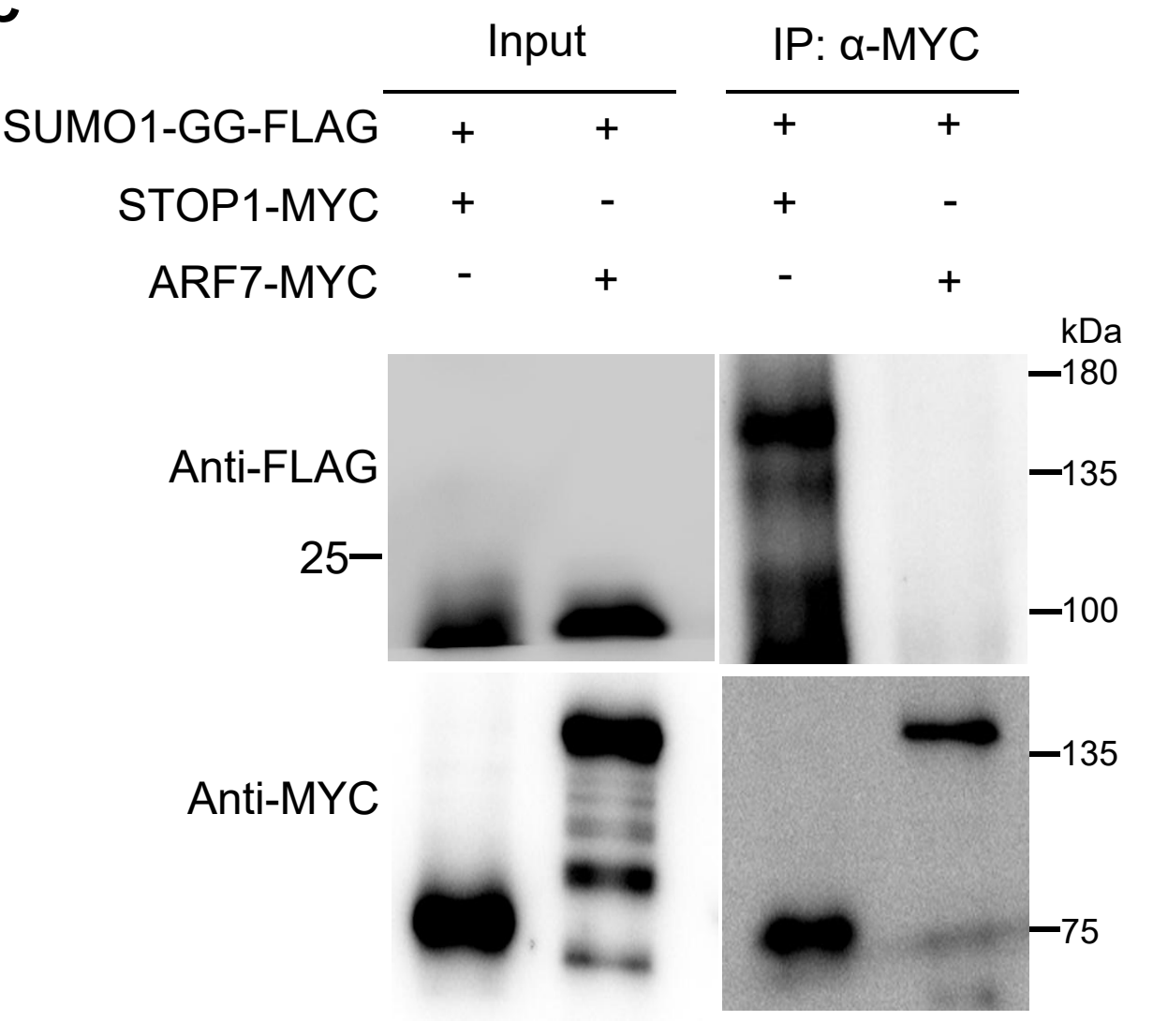

Figure S10 SIZ1 interacts with ARF7

(A) The sequence intensity of ARF7 identified by IP-MS using 10-d-old *pro35S:SIZ1-GFP* and *pro35S:GFP* seedling roots.
(B) Y2H assay for detecting the interaction between SIZ1 and ARF7. Yeast cells co-transformed *pGADT7-ARF7* and *pGBKT7-SIZ1* were dropped onto SD-WL and SD-WLAH + X-α-gal + 3 mM 3-AT medium. The negative control and positive control were transformed with commercial plasmids. AD, activation domain; BD, DNA binding domain.
(C) ARF7 SUMOylation detected in Col-0 protoplasts. *ARF7-MYC* was transiently co-expressed with *FLAG-SUMO1-GG* in Col-0 protoplasts. Total proteins extracts (input) were immunoprecipitated with anti-MYC antibody. Anti-MYC and anti-FLAG antibodies were used for the immunoblotting analyses of the input proteins and the immunoprecipitated proteins. *STOP1-MYC* was positive control.

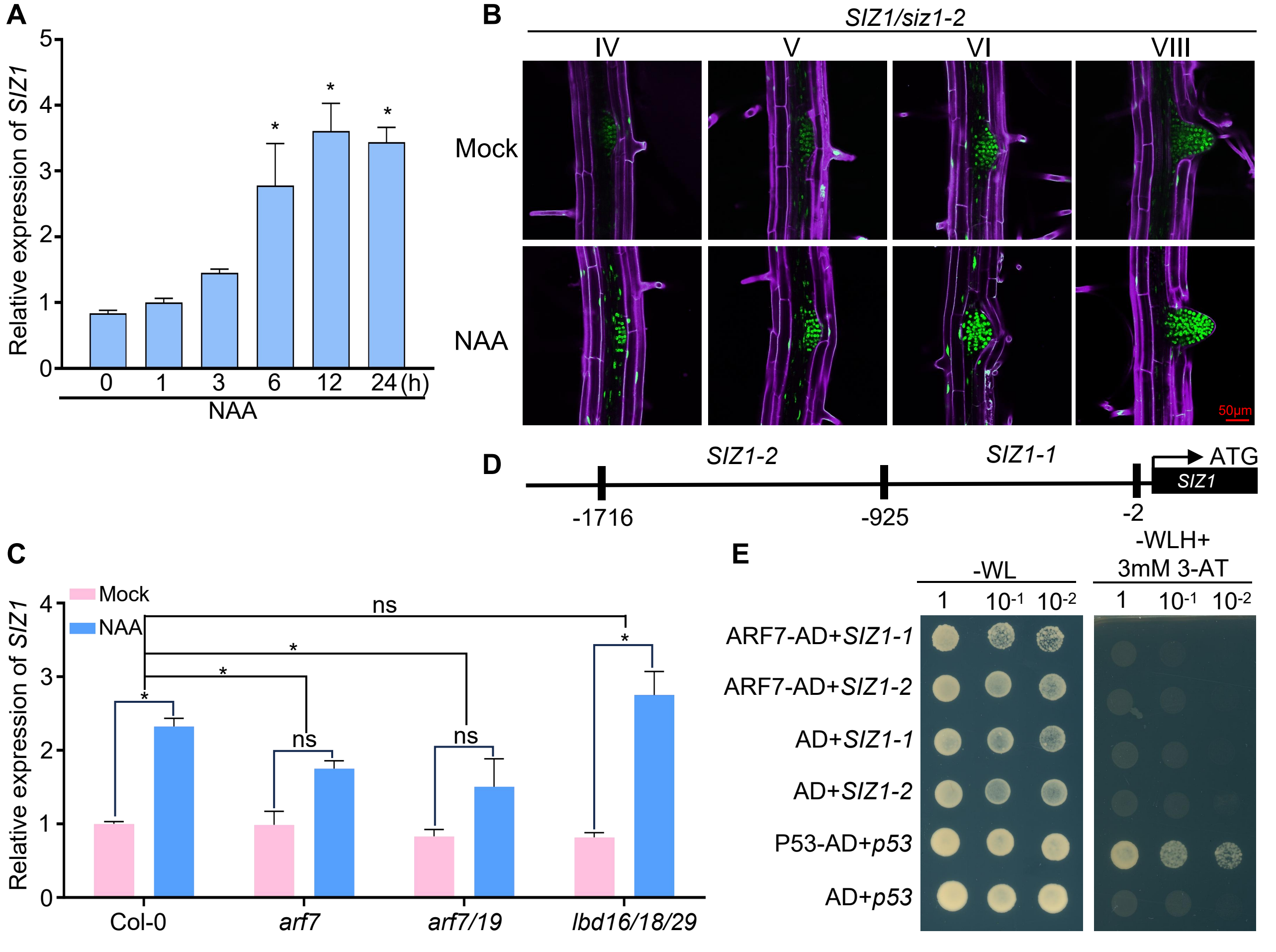

Figure S11 NAA induces *SIZ1* expression

(A) Real-time fluorescence quantitative PCR (qRT-PCR) analysis showing the relative expression levels of *SIZ1* in the Col-0 with or without 10 μM NAA. Total RNA was extracted from primary roots of 8-d-old seedlings. Three biological replicates with three technical replicates for each biological replicate were performed with similar results. Data represent mean ± SE ($n = 3$). *$P < 0.05$, as determined by a Student's *t* test.
(B) Fluorescence images of the LRP stage VI and VIII of 8-d-old *ProSIZ1:SIZ1/siz1-2* seedlings treated with or without 10 μM NAA for 12 h. In these fluorescent images, we replaced red (propidium iodide signal) with magenta. Bar, 50 μm.
(C) qRT-PCR analysis showing the relative expression levels of *SIZ1* in the Col-0, *arf7*, *arf7/19*, and *lbd16/18/29* treated with or without 10 μM NAA. Total RNA was extracted from primary roots of 8-d-old seedlings. The expression level in the Col-0 was set to "1." Three biological replicates with three technical replicates for each biological replicate were performed with similar results. Data represent mean ± SE （$n = 3$). *$P < 0.05$, as determined by a Student's *t* test.
(D) Schematic diagram of the *SIZ1* promoter used for Y1H assay.
(E) Y1H assay testing binding of ARF7 to the *SIZ1* promoter. The yeast transformants were dropped onto SD-WL medium. The yeast transformants were dropped onto SD-WLH + 3 mM 3-AT medium. The negative control and positive control were transformed with commercial plasmids.

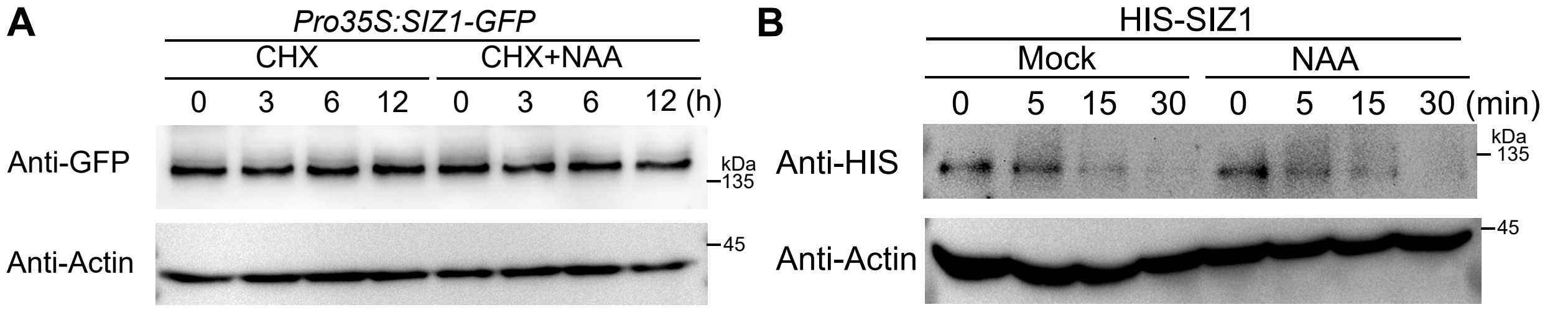

Figure S12 Auxin does not affect the protein stability of SIZ1

(A) *In vivo* assay for SIZ1 protein stability. 10-d-old seedlings of *Pro35S:SIZ1-GFP* were subjected to treatments with 150 μM CHX or a combination of 150 μM CHX + 10 μM NAA for 0, 3, 6 and 12 h, respectively. SIZ1 was detected by anti-GFP antibody. Actin served as a loading control.

(B) Cell-free assays showed that auxin does not affect the degradation of SIZ1 *in vitro*. Total proteins were extracted from 10-d-old seedlings of WT with or without 10 μM NAA and incubated with recombinant HIS-SIZ1 for 0, 5, 15 and 30 min, respectively. HIS-SIZ1 protein was detected by anti-HIS antibody. Actin served as a loading control.

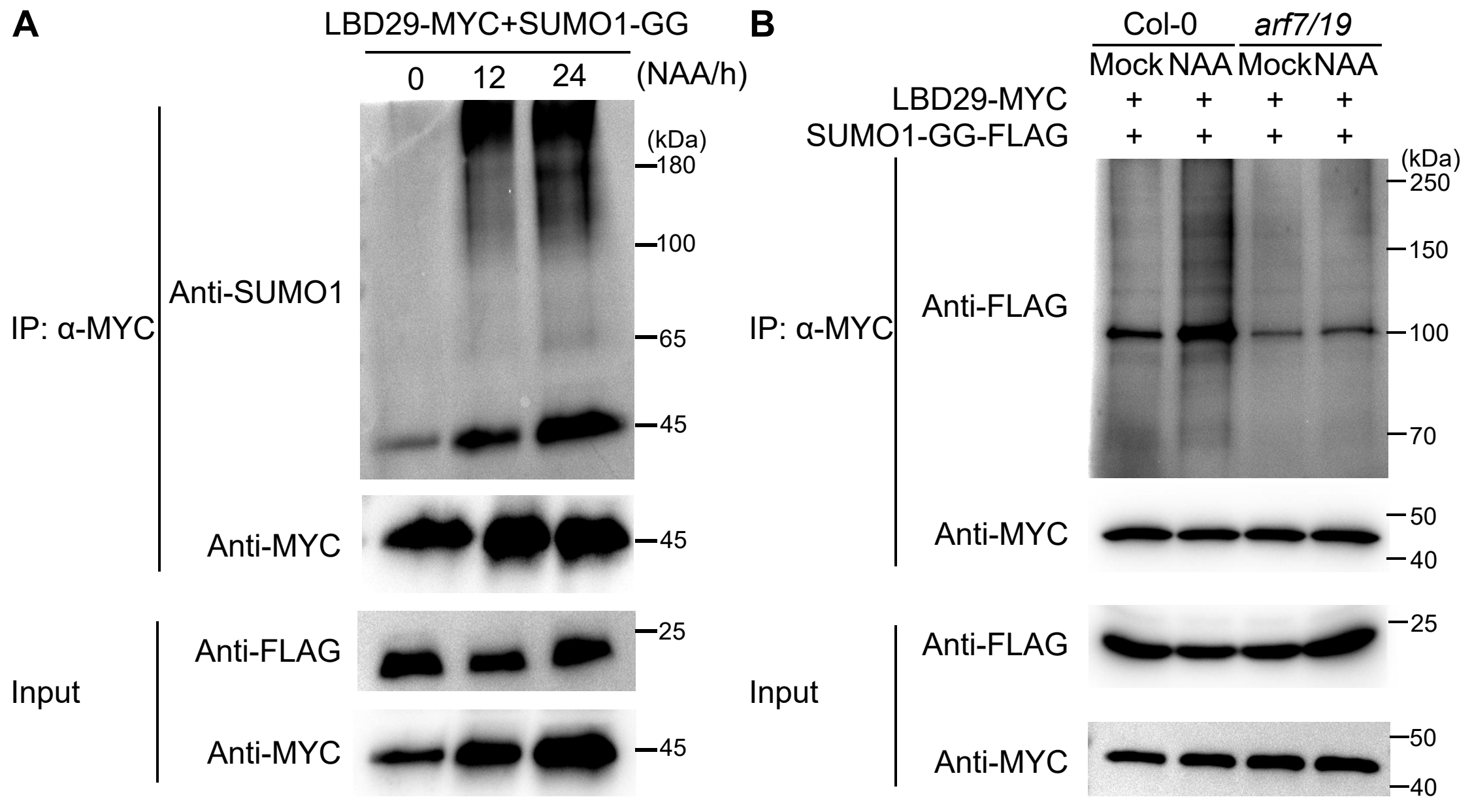

Figure S13 Auxin-induced SUMOylation of LBD29 is dependent on *ARF7/19*

(A) co-IP assays performed in *N. benthamiana* leaves to validate the effect of NAA on LBD29 SUMOylation. The constructs *LBD29-MYC* and *SUMO1-FLAG* were co-transformed into *N. benthamiana* leaves. After 12 h of dark incubation followed by 48 h under normal light conditions, the leaves were treated with 10 μM NAA 0 h, 12 h, 24 h. Proteins were immunoprecipitated using an anti-MYC antibody, and SUMOylation of LBD29 was detected by immunoblotting with an anti-SUMO1 antibody.
(B) LBD29-MYC was transiently co-expressed with FLAG- SUMO1-GG in WT and *arf7/19* protoplasts. After overnight incubation, the protoplasts were treated with 100 nM NAA for 4 h. Total proteins extracts (input) were immunoprecipitated with anti-MYC antibody. Anti-MYC and anti-FLAG antibodies were used for the immunoblotting analyses of the input proteins and the immunoprecipitated proteins.
